# Multifunctional Carbon Foam with Nanoscale Chiral Magnetic Heterostructures for Broadband Microwave Absorption in Low Frequency

**DOI:** 10.1007/s40820-025-01658-8

**Published:** 2025-02-06

**Authors:** Hao Zhang, Kaili Kuang, Yifeng Zhang, Chen Sun, Tingkang Yuan, Ruilin Yin, Zeng Fan, Renchao Che, Lujun Pan

**Affiliations:** 1 School of Physics, Dalian University of Technology, Dalian, 116024 Liaoning People’s Republic of China; 2 Laboratory of Advanced Materials, Shanghai Key Lab of Molecular Catalysis and Innovative Materials, Department of Materials Science, Fudan University, Shanghai, 200438 People’s Republic of China

**Keywords:** Carbon nanocoils, Chiral magnetic structures, 3D conductive networks, Magnetic pinning effect, Broadband microwave absorption

## Abstract

The construction of carbon nanocoil (CNC)-based chiral-dielectric-magnetic trinity composites is considered as a promising approach to achieve excellent low-frequency microwave absorption. However, it is still challenging to further enhance the low frequency microwave absorption and elucidate the related loss mechanisms. Herein, the chiral CNCs are first synthesized on a three-dimensional (3D) carbon foam and then combined with the FeNi/NiFe_2_O_4_ nanoparticles to form a novel chiral-dielectric-magnetic trinity foam. The 3D porous CNC-carbon foam network provides excellent impedance matching and strong conduction loss. The formation of the FeNi-carbon interfaces induces interfacial polarization loss, which is confirmed by the density functional theory calculations. Further permeability analysis and the micromagnetic simulation indicate that the nanoscale chiral magnetic heterostructures achieve magnetic pinning and coupling effects, which enhance the magnetic anisotropy and magnetic loss capability. Owing to the synergistic effect between dielectricity, chirality, and magnetism, the trinity composite foam exhibits excellent microwave absorption performance with an ultrabroad effective absorption bandwidth (EAB) of 14 GHz and a minimum reflection of loss less than − 50 dB. More importantly, the C-band EAB of the foam is extended to 4 GHz, achieving the full C-band coverage. This study provides further guidelines for the microstructure design of the chiral-dielectric-magnetic trinity composites to achieve broadband microwave absorption.

**Supplementary Information:**

The online version contains supplementary material available at 10.1007/s40820-025-01658-8.

## Highlights

- A novel multifunctional carbon foam with nanoscale chiral magnetic heterostructures is constructed, in which the interconnection network provides strong conduction loss.
- The interfacial polarization loss induced by the FeNi-carbon interfaces is confirmed by the density functional theory calculations, and the magnetic pinning and coupling effects are revealed by the micromagnetic simulation.
- The composite foam exhibits an ultrabroad effective absorption bandwidth (EAB) of 14 GHz and a C-band EAB of 4 GHz, achieving the full C-band coverage.

**Supplementary Information:**

The online version contains supplementary material available at 10.1007/s40820-025-01658-8.

## Graphical abstract

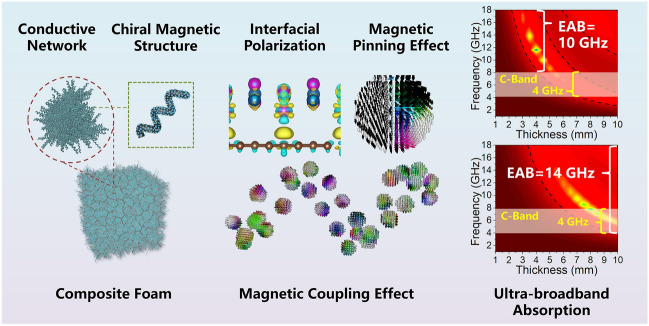

## Introduction

Electronic devices and systems based on the fifth-generation (5G) technologies bring great convenience to daily life. However, the electromagnetic wave interference/radiation is also becoming a potential threat [1–3]. Multitudinous microwave absorption materials with excellent absorption performance have been developed, most of which exhibit wide effective absorption bandwidth (EAB) in the high microwave frequency region (10–18 GHz) [4–7]. Nevertheless, the 5G technologies signals fall in the low microwave frequency region (2–10 GHz), especially in the C-band (4–8 GHz). Therefore, further expanding the low frequency absorption bandwidth of the microwave absorption materials is critical but remains challenging.

It is anticipated that magnetic materials with superior natural resonance [8] and exchange resonance [9] would be effective in achieving excellent low-frequency microwave absorption, owing to their strong magnetic loss ability. However, it is difficult to further improve the magnetic loss ability of the magnetic materials due to the Snoke’s limit [10]. Geometric regulation, particularly the construction of magnetic anisotropic assemblies, is an efficient strategy for promoting the Snoke’s limit. For example, Che et al. designed the nonsymmetric hammer-shaped Fe/Fe_3_O_4_@SiO_2_ composite, achieving strong magnetic loss ability [11]. The nonsymmetric distribution of the magnetic components is beneficial to enhance the magnetic anisotropy, which further promotes the Snoke’s limit. Moreover, the magnetic anisotropy is also affected by the compositional discrepancy of the magnetic components. Magnetic heterostructures, especially at the nanoscale, have great potential for enhancing the magnetic anisotropy. Wang et al. constructed the ferromagnetic/antiferromagnetic heterostructures, confirming that magnetic pinning effect induced by the interfacial magnetic bias improves the magnetic anisotropy and low frequency permeability [12]. Therefore, achieving the nonsymmetric distribution of the nanoscale magnetic heterostructures could be an effective strategy to promote the Snoke’s limit. Furthermore, the high density of the magnetic materials results in a high filling ratio in the absorbers, which is disadvantageous for their applications. It is also important to combine magnetic materials with a lightweight dielectric material that can simultaneously achieve strong magnetic loss and low density.

Carbon nanocoils (CNCs) have been considered as a kind of versatile nanomaterial in many fields [13–27]. Compared to other carbon materials such as graphene, carbon nanotubes and carbon nanofibers, the CNCs have moderate conductivity, which is beneficial for adjusting impedance matching. The point-to-point contact between CNCs also prevents them from aggregation to form a uniformly dispersed composite. More importantly, the CNCs possess obvious 3D helical morphology, which provides the excellent chiral template for magnetic components. The nanoscale line diameter and the helical structure of the CNCs could contribute to the confined space synthesis of the magnetic heterostructures. Besides, the unique chiral morphology of the CNCs could also give rise to the nonsymmetric distribution of the magnetic heterostructures. Therefore, the CNCs are considered as the excellent template materials for construction of the chiral magnetic absorbers. Although various magnetic/CNC composites, including Fe_3_O_4_/Al_2_O_3_/CNC [28], FeCo@FeCo_2_O_4_/CNC [29], and CoNi/CNC [30], have been developed and achieved excellent microwave absorption performance, the EAB in the low frequency range still requires further extension. The internal mechanisms of enhanced magnetic loss also remain to be investigated. In addition, most of the current magnetic/CNC composites are in the form of powder, which require additional support matrix and exhibit narrow band impedance matching. It is believed that the three-dimensional (3D) porous structures such as foams and aerogels tend to broaden the impedance matching range [31–33]. In the porous structures, the presence of air reduces the permittivity, which contributes to the excellent impedance matching. Meanwhile, the efficient interconnected networks ensure the conduction loss and avoid the agglomeration. Therefore, if the chiral magnetic units are uniformly arranged in a 3D porous interconnected network, the microwave absorption performance would be improved significantly.

In this work, the chiral CNCs are first synthesized on a three-dimensional (3D) carbon foam and then combined with the FeNi/NiFe_2_O_4_ nanoparticles to form a novel chiral-dielectric-magnetic trinity foam via chemical vapor deposition (CVD) and solvothermal reactions. The porous CNC-carbon foam skeleton forms an 3D interconnected conductive network. The nanoscale FeNi/NiFe_2_O_4_ magnetic heterostructures, in which the FeNi acts as the ferromagnetic component while the NiFe_2_O_4_ acts as the ferrimagnetic component, are uniformly synthesized on the chiral CNC. The FeNi/carbon interfaces contribute to the interfacial polarization loss. Meanwhile, the formation and nonsymmetric distribution of the nanoscale magnetic heterostructures lead to the magnetic pinning and coupling effect, which promotes the Snoke's limit and increases the magnetic loss. With the synergistic effect between chirality, magnetism and dielectricity, the composite carbon foam exhibits superior microwave absorption performance in both low and high frequencies.

## Experimental Section

### Materials

Deionized water, ethanol, and nickel nitrate (Ni(NO_3_)_2_·6H_2_O) were purchased from Tianjin Kermel Chemical Reagent Co., Ltd. Ferrous sulfate (FeSO_4_·7H_2_O) and hexamethylenetetramine (HMT) were purchased from Shanghai Sinopharm Chemical Reagent Co., Ltd. Melamine foam was provided by Shanghai Junhua Hightech Materials Co., Ltd. All chemical reagents were of analytical grade and were used without further purification.

### Preparation of CF, CCF, FCF, and FCCF Composites

#### Preparation of Carbon Foam (CF)

The carbon foam samples were obtained through the carbonization of the melamine foams. Typically, the melamine foams were placed in a tube furnace (BTF-1200C-III-S, Anhui BEQ) under an Ar atmosphere of 492.5 sccm. The foam samples were heated to 700 °C at a heating rate of 5 °C min^−1^ and then carbonized at 700 °C for two hours. Finally, the carbon foams were collected and labeled as CF.

#### Preparation of Carbon Nanocoil/Carbon Foam (CCF) Composites

**Fig. 1:**
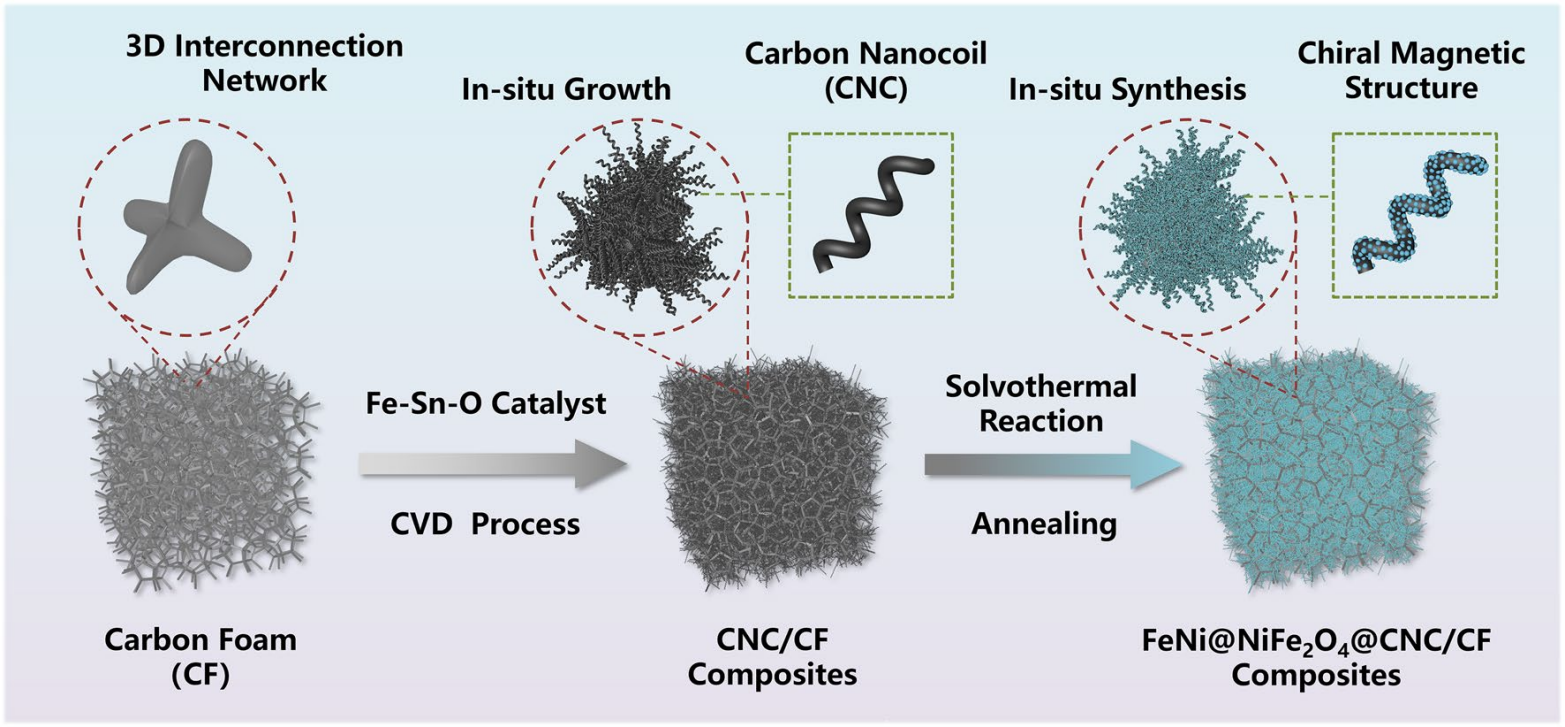
Schematic illustration of synthesis process for the CCF and FCCF samples

As shown in Fig. 1, the CNC/CF composites were prepared by the CVD process. Briefly, the Fe-Sn–O catalyst was prepared according to our previous research [34, 35] and dispersed in ethanol. The as-prepared CF samples were immersed in the Fe-Sn–O catalyst dispersion and dried at 60 °C for 2 h. Then, the CF samples coated with catalyst were placed in the tube furnace under 563.2 sccm Ar atmosphere. Finally, the CNCs were synthesized on the CF samples with the introduction of additional 23.24 sccm C_2_H_2_ gases at 710 °C and the CNC/CF (denoted as CCF) composites were obtained.

#### Preparation of FeNi@NiFe_2_O_4_@CNC/CF (FCCF) and FeNi@NiFe_2_O_4_/CF (FCF) Composites

Generally, 2 mmol Ni(NO_3_)_2_·6H_2_O, 1 mmol FeSO_4_·7H_2_O, and 4 mmol HMT were added into 40 mL mixed solvent of ethanol and deionized water (volume ratio was 1:1). The mixture was then stirred for 20 min to form the precursor solution. The as-prepared CCF composites were functionalized by the plasma treatment for 10 min. The functionalized CCF composites and the precursor solution were transferred into a 50-mL Teflon-lined autoclave and then subjected to a solvothermal reaction at 100 °C for 10 h. After the reaction, the FeNi@NiFe_2_O_4_@CNC/CF precursors were washed with deionized water and dried at 60 °C overnight. According to our previous research [29], an annealing temperature of 700 °C is chosen to ensure the formation of the FeNi/NiFe_2_O_4_ magnetic heterostructures. Finally, the FeNi@NiFe_2_O_4_@CNC/CF precursors were annealed at 700 °C for 2 h under 492.5 sccm Ar atmosphere to prepare the FeNi@NiFe_2_O_4_@CNC/CF composites, labeled as FCCF-1. Repeatedly, the precursor solutions were used at 1.5 times and twice the concentration to prepare two other FeNi@NiFe_2_O_4_@CNC/CF composites, denoted as FCCF-2 and FCCF-3, respectively. All other conditions remained unchanged. For comparison, the FeNi@NiFe_2_O_4_/CF composites (denoted as FCF) were also prepared by changing the CCF composites as pure CF samples. All other conditions were kept unchanged.

### Characterization

Morphological and microstructural information of the samples was obtained using a field emission scanning electron microscopy (SEM, SEM5000, CIQTEK Co., Ltd.) and a transmission electron microscopy (TEM, JEM F-200, JEOL). X-ray diffraction (XRD, Lab XRD-7000 s) with a Cu Kα radiation source was used to characterize the crystal structure of the composites. Raman spectroscopy (Finder930, Zolix) was used to study the chemical states of the composites. The magnetic properties of the samples were obtained using a vibrating sample magnetometer (VSM, LakeShore 7400S). The electromagnetic parameters were measured using a vector network analysis (VNA, Keysight E5080B) in the frequency range of 1–18 GHz. The composites were cut into a coaxial ring shape with an external diameter of 7.00 mm, an internal diameter of 3.04 mm and a thickness of 2.0 mm. The prepared coaxial ring samples were then used directly for the measurement of the electromagnetic parameters without any supporting matrix.

## Results and Discussion

### Morphology and Structure Information of the Samples

**Fig. 2:**
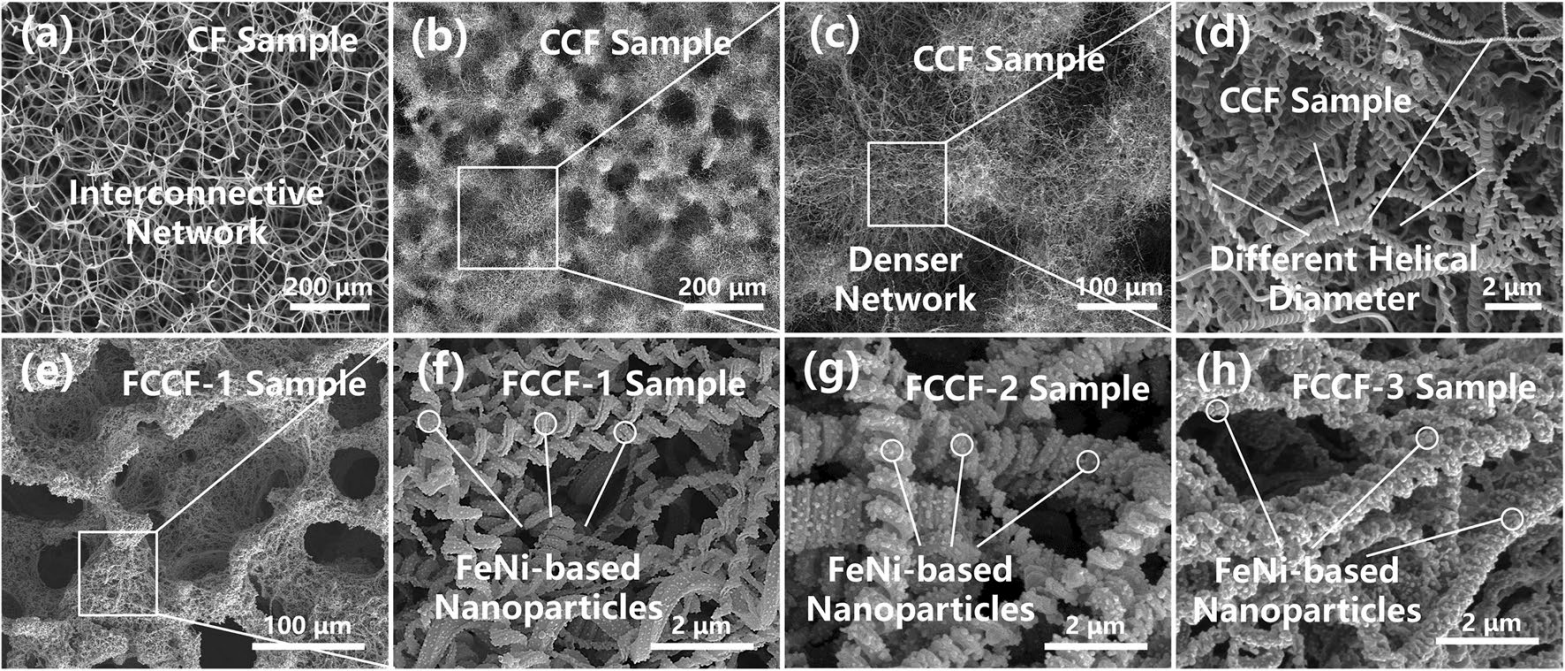
SEM images of **a** CF sample, **b–d** CCF sample, **e, f** FCCF-1 sample, **g** FCCF-2 sample, and **h** FCCF-3 sample

The microstructures and morphologies of the as-prepared samples are depicted in the SEM images (Figs. 2 and S1). The initial CF sample exhibits a typical 3D interconnecting network formed by the carbon fibers with the diameter of about 5 μm (Figs. 2a and S1a). It is considered that the 3D interconnective skeleton has been successfully established by the carbon foam. As shown in Fig. 2b, c, lots of CNCs grow uniformly on the carbon foam skeleton after the CVD process. The CF-CNC structures form a denser interconnection network, which further enhances the electron transfer capability of the composite. In addition, the formation of the CF-CNC structures also improves the specific surface area of the composite, which gives rise to the multiple scattering of the microwave. In Fig. 2d, it is observed that the CNCs exhibit an obvious chiral helical morphology, which provide the excellent chiral templates for the fabrication of the chiral magnetic structures. Moreover, the helical diameter of the synthesized CNCs varies from 100 to 500 nm, which is beneficial for the construction of the multi-scale composite. It is believed that the multi-scale composites tend to achieve multi-band absorption effect, which is beneficial for further expanding the EAB of the absorbers. Figure 2e shows that the 3D interconnection network could be well maintained during the solvothermal reaction and the annealing treatment. Furthermore, the FeNi-based magnetic nanoparticles are synthesized uniformly on the surface of the CNCs, forming the chiral magnetic structures (Fig. 2f–h). Furthermore, it is also observed in Fig. 2f–h that the growth density and size of the FeNi-based nanoparticles increase gradually as the concentration of the precursor solution rises. However, without the CNCs, the FeNi-based particles would grow on the carbon fiber skeleton directly with a diameter of around 200–500 nm, which tend to aggregate with each other (Fig. S1b–d). Therefore, it is reasonable to conclude that the curved surface and the small line diameter endow the CNCs with the confinement effect, which not only contributes to the formation of the nanoscale FeNi-based particles, but also prevents the nanoparticles from aggregation.

**Fig. 3:**
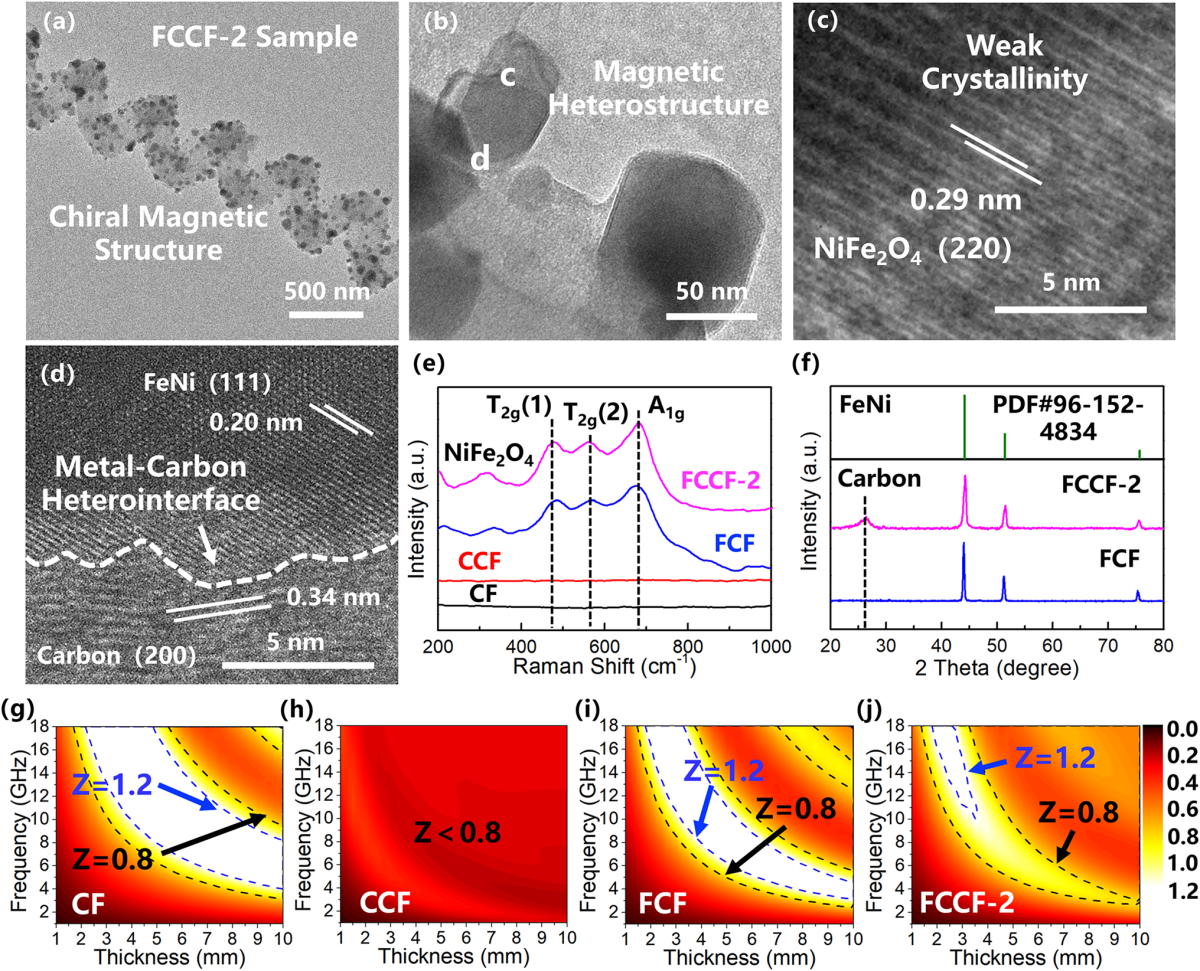
**a, b** TEM and **c, d** HRTEM images of the FCCF-2 composites; **e** Raman spectra of the CF, CCF, FCF, and FCCF-2 composites; **f** XRD patterns of the FCF and FCCF-2 composites; Impedance matching contour maps of **g** CF, **h** CCF, **i** FCF, and **j** FCCF-2 samples

The morphology and structural information of the FeNi@CNC units in the FCCF-2 sample are further shown in the TEM images (Fig. 3a–d). The FeNi@CNC unit shows an obvious chiral structure with the uniform distribution of the FeNi-based nanoparticles (Fig. 3a, b), which is consistent with the SEM results. Besides, the HR-TEM image of the internal part of the nanoparticle (Fig. 3c) exhibits the lattice fringes with an interplanar spacing of 0.29 nm, corresponding to the (220) crystal plane of NiFe_2_O_4_. The relatively blurred lattice fringes reveal the weak crystallinity of the NiFe_2_O_4_. It could therefore be surmised that additional reactions may occur during the annealing treatment, resulting in the destruction of the lattice structure of the NiFe_2_O_4_. Furthermore, the HR-TEM image of the nanoparticle surface (Fig. 3d) shows a lattice fringe with an interplanar spacing of 0.20 nm, corresponding to the (111) crystal plane of FeNi. Moreover, another crystalline structure is also observed in Fig. 3d. The interplanar spacing is measured as 0.34 nm, corresponding to the (002) crystal plane of graphite carbon. Therefore, during the annealing treatment, the NiFe_2_O_4_ is partially reduced to the metal FeNi by the carbon due to the carburization effect [36], resulting in the formation of the FeNi/NiFe_2_O_4_ magnetic heterostructure and the metal–carbon heterointerface. The Raman spectra of the samples were tested to confirm this conclusion. As shown in Fig. 3e, the CF and CCF samples do not exhibit any peak in the 0–1000 cm^−1^ region. For the FCCF and FCF composites, the introduction of the FeNi-based particles results in the appearance of three main peaks appearing at 480, 570, and 683 cm^−1^, which correspond to the T_2g_(1), T_2g_(2), and A_1g_ vibrational modes of the NiFe_2_O_4_ [37, 38]. The Raman results further confirm the existence of the NiFe_2_O_4_ in the FeNi-based particles, which is consistent with the TEM images. Moreover, the XRD patterns of the FCCF and FCF samples (Fig. 3f) provide further structural information of the FeNi-based particles. It is observed that the FCCF and FCF samples exhibit three obvious diffraction peaks at 44.1°, 51.4°, and 75.7°, corresponding to the (111), (020), and (022) crystal planes of the metal FeNi (JCPDS No. 96-152-4834). The sharp diffraction peaks indicate that the metal FeNi possesses good crystallinity, which is consistent with Fig. 3d. It is also worth noting that the XRD patterns do not exhibit any characteristic peak of the NiFe_2_O_4_, confirming that the NiFe_2_O_4_ in the FeNi-based particles possesses weak crystallinity (Fig. 3c). In addition, a prominent peak at 25.6° appears in the XRD pattern of the FCCF-2 sample, corresponding to the (002) crystal plane of graphitic carbon. It is confirmed that the synthesis of the CNCs improves the electron transfer capability of the carbon-based network. As illustrated in Fig. S2a, b, the XRD and Raman spectra of the FCCF-1 and FCCF-3 samples exhibit a high degree of consistency with the XRD and Raman spectra of the FCCF-2 sample, indicating that the FeNi/NiFe_2_O_4_ magnetic heterostructures are also synthesized in the FCCF-1 and FCCF-3 samples. In a word, the 3D interconnection network with the chiral magnetic units has been successfully established, exhibiting excellent electron transfer capability and a multitude of FeNi/NiFe_2_O_4_ magnetic heterostructures.

Impedance matching (*Z*) is considered the primary factor for excellent microwave absorbers, since the *Z* represents the ability of microwaves to enter the interior of the absorber [39–42]. The *Z* value is calculated according to the following formula [43–47]:

1 $$ Z = \frac{{Z_{{{\text{in}}}} }}{{Z_{0} }}{ = }\sqrt {\left| {\frac{{\mu_{{\text{r}}} }}{{\varepsilon_{{\text{r}}} }}} \right|} \tanh \left[ {j\left( {{{2\pi fd} \mathord{\left/ {\vphantom {{2\pi fd} c}} \right. \kern-0pt} c}} \right)\sqrt {\mu_{{\text{r}}} \varepsilon_{{\text{r}}} } } \right] $$

where $Z_{{{\text{in}}}}$ and $Z_{0}$ refer to the input impedance of the microwave absorber and the free space, respectively; *f* represents the frequency of microwave; *d* is the thickness of the microwave absorber; and *c* stands for the velocity of light. It is observed in the formula that the *Z* value is closely related to the complex permittivity ($\varepsilon_{{\text{r}}} = \varepsilon^{\prime } - j\varepsilon^{\prime \prime }$) and permeability ($\varepsilon_{{\text{r}}} = \varepsilon^{\prime } - j\varepsilon^{\prime \prime }$), in which the real parts ($\varepsilon^{\prime }$ and $\mu^{\prime }$) refer to the electric and magnetic energy storage capacities, while the imaginary parts ($\varepsilon^{\prime \prime }$ and $\mu^{\prime \prime }$) represent the energy dissipation capacities [48, 49]. Thus, the electromagnetic parameters of the samples are tested (Fig. S3) and the *Z* values of the samples are further calculated (Figs. 3g-j and S4). Generally, a *Z* value of 1 means that all the microwaves could enter the interior of the absorber without reflection. Accordingly, a *Z* value between 0.8 and 1.2 (close to 1) is essential to produce superior microwave absorbers. In Fig. 3g, h, the impedance matching of the CF and CCF is poor due to their inadequate or excessive permittivity value. For the FCF sample, the introduction of the FeNi/NiFe_2_O_4_ particles modulates the permittivity, which promotes the impedance matching in some degree (Fig. 3i). Furthermore, Figs. 3j and S4 illustrate that the impedance matching of the FCCF composites is greatly promoted by the introduction of the CNCs and FeNi/NiFe_2_O_4_ nanoparticles. Among the samples, the FCCF-2 exhibits the optimal impedance matching. Therefore, it is concluded that the CNCs and FeNi-based nanoparticles effectively collaborate with each other in permittivity modulation, contributing to good impedance matching.

### Microwave Absorption Performance and Multifunctional Properties of the Samples

The RL value is the most used index for evaluating the microwave absorption performance of absorbers. According to the transmission line theory, the formula for calculating the RL value is as follows [50–52]:

2 $$ RL(dB) = 20\log \left| {{{\left( {Z_{{{\text{in}}}} - Z_{0} } \right)} \mathord{\left/ {\vphantom {{\left( {Z_{{{\text{in}}}} - Z_{0} } \right)} {\left( {Z_{{{\text{in}}}} + Z_{0} } \right)}}} \right. \kern-0pt} {\left( {Z_{{{\text{in}}}} + Z_{0} } \right)}}} \right| $$

**Fig. 4:**
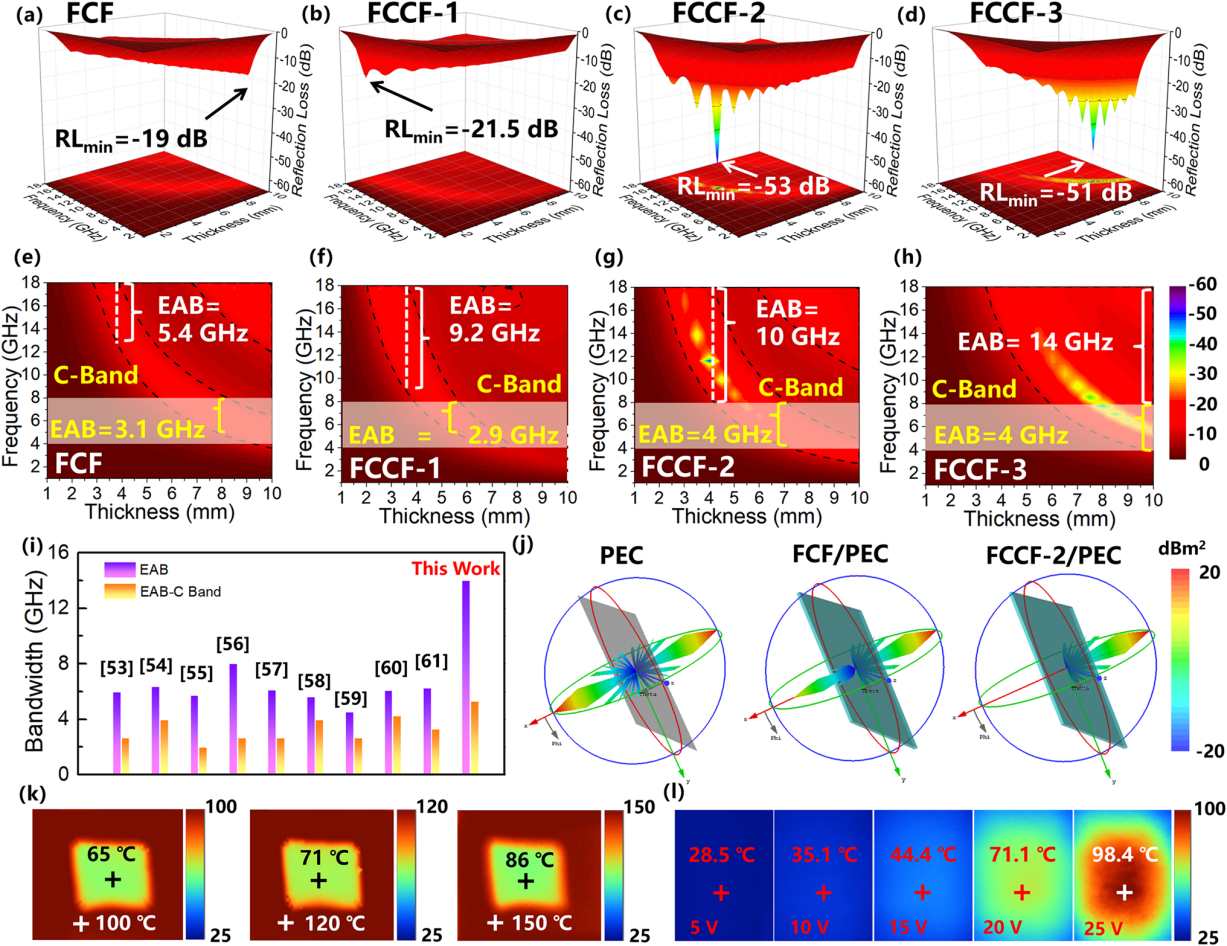
3D RL values and their projection plots of **a, e** CF, **b, f** CCF, **c, g** FCF, and **d, h** FCCF-2 samples; **i** comparison of the maximum EAB and the maximum C-Band EAB values with other aerogel/foam-based absorbers reported recently in literature; **j** 3D RCS plots for PEC substrate, PEC substrate covered with FCF and FCCF-2; **k** Infrared radiation images of surface temperature of FCCF-2 sample on a platform with different temperatures; **l** Infrared radiation images of surface temperature of FCCF-2 sample under different driving voltages

As shown in Fig. S5, the CF (Fig. S5a, c) and CCF (Fig. S5b, d) samples exhibit low RL values and inadequate microwave absorption performance, which can be attributed to the suboptimal impedance matching. For the FCF sample (Fig. 4a, e), the microwave absorption performance gets better in some degree because of the promoted impedance matching. The minimum *RL* reaches -19 dB, and the maximum EAB is 5.4 GHz. It is also observed in Fig. 4g that the EAB value in C-band reaches 3.1 GHz, confirming that the 3D magnetic foam structure has potential in low frequency microwave absorption. Furthermore, the FCCF-1 sample (Fig. 4b, f) achieves a broad EAB of 9.2 GHz with a thickness of 3.5 mm, indicating that the chiral magnetic units are beneficial for extending the EAB. The C-band EAB of the FCCF-1 sample reaches 2.9 GHz. In addition, the FCCF-2 sample (Fig. 4c, g), with a higher density of magnetic nanoparticles, shows a wider EAB of 10 GHz with a thickness of 4 mm. More importantly, the C-band EAB of the FCCF-2 sample is also expanded to 4 GHz, achieving the full C-band coverage. With the further increase in the growth density of the magnetic nanoparticles, the FCCF-3 sample (Fig. 4d, h) achieves an ultrabroad EAB of 14 GHz. However, the corresponding thickness rises to 10 mm, which is much thicker than the FCCF-2 sample. In a word, the FCCF samples all exhibit superior microwave absorption performance compared with the FCF sample. On the one hand, the excellent microwave absorption performance of the FCCF samples is attributed to their better impedance matching. On the other hand, the introduction of the CNCs and the magnetic heterostructures further enhances the dielectric and magnetic losses. As shown in Fig. 4i, it is concluded that the FCCF samples achieve both high EAB and C-band EAB values compared to the carbon-based 3D aerogel/foam absorbers in other studies [53–61]. Therefore, the FCCF samples could exhibit excellent microwave absorption performance in both low frequency and high frequency regions.

**Fig. 5:**
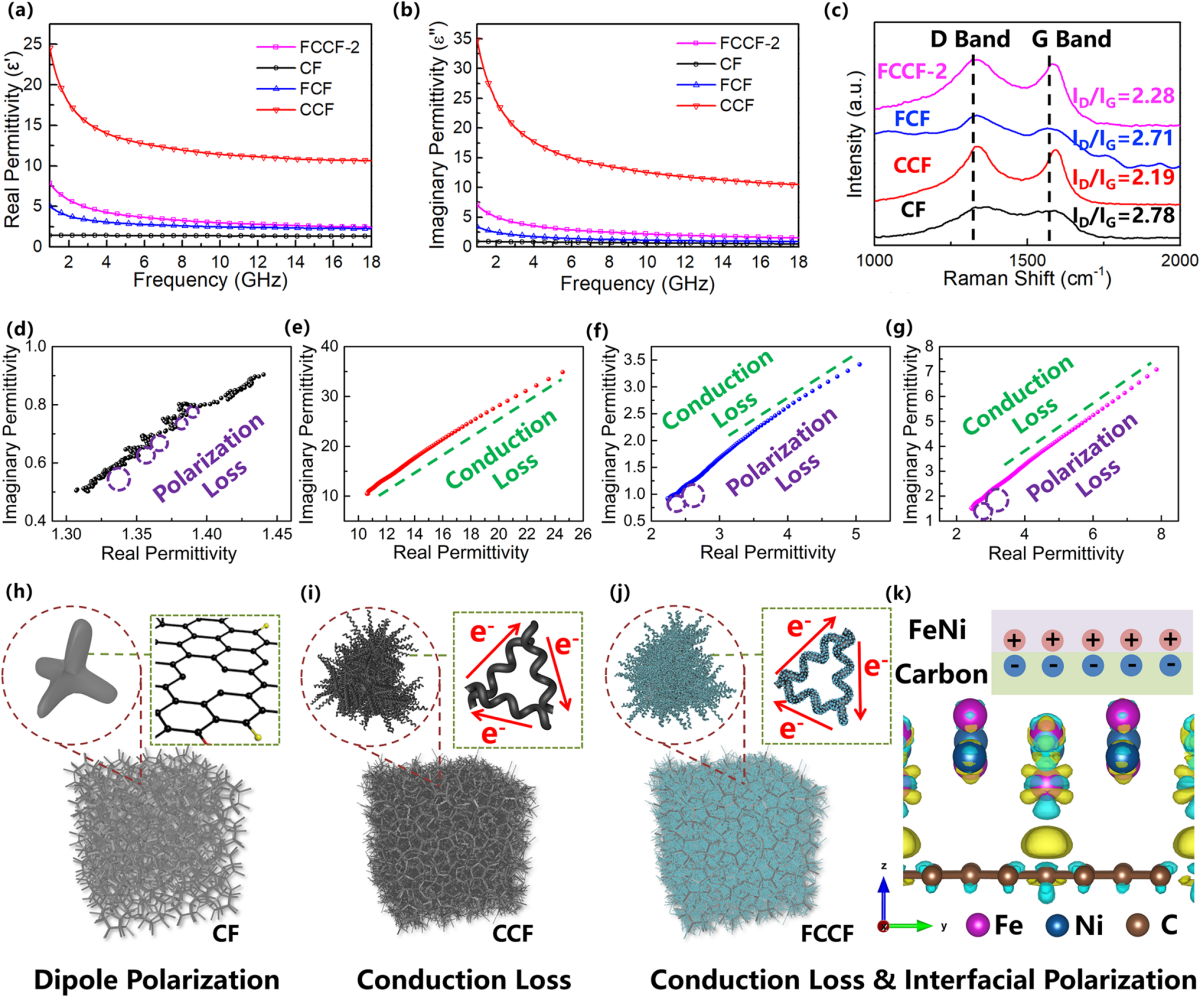
**a** Real permittivity, **b** imaginary permittivity, and **c** Raman spectra of CF, CCF, FCF, and FCCF-2 samples; Cole–Cole plots of **d** CF, **e** CCF, **f** FCF, and **g** FCCF-2 samples; Dielectric loss diagram of **h** CF, **i** CCF, and **j** FCCF samples; **k** Calculated electron density difference and corresponding isosurface plot. Electron density difference was calculated by Δ = ρ(C/FeNi)-ρ(C)-ρ(FeNi). The yellow and cyan regions denote electron density accumulation and depletion, respectively

To further evaluate the actual far-field microwave absorption performance of the FCCF samples in real situations, radar cross section (RCS) simulations are performed based on the electromagnetic parameters. In general, theta and phi in spherical coordinates determine the scattering direction of the RCS value ($\sigma$) for a given scattering source as follows [62–65]:

3 $$ \sigma \left( {{\text{dBm}}^{{2}} } \right) = 10\log \left[ {\frac{4\pi S}{{\lambda^{2} }}\left| {\frac{{E_{S} }}{{E_{i} }}} \right|^{2} } \right] $$

where $E_{S}$ and $E_{i}$ refer to the electric field intensities of the accepting wave; $\lambda$ represents the wavelength of the incident microwave; and *S* stands for the area of the simulated plate. The RCS is the parameter used to quantify the echo intensity of a target material when illuminated by radar waves. Consequently, a weaker RCS signal indicates a greater microwave absorption capability of the sample. In this study, RCS simulations are performed using the CST STUDIO SUITE 2019 software, with the positive x-axis set as the direction of the microwave. As shown in Fig. 5j, the pristine perfect electric conductor (PEC) plate exhibits strong RCS scattering signal, indicating weak microwave absorption capability. However, the PEC plate coated with the FCF sample shows lower scattering signal due to the microwave absorption capability of the FCF sample. Furthermore, the FCCF-2/PEC model exhibits the lowest scattering signal, revealing that the FCCF-2 sample possesses the strongest microwave absorption capability. Due to the consistency of the measured microwave absorption results and the RCS simulation results, it is reasonable to conclude that the FCCF-2 composites show great potential for practical applications as excellent microwave absorbers.

In addition to the microwave absorption performance, the FCCF-2 composites also possess multifunctional properties. As shown in Fig. S6a, the FCCF-2 sample could easily be perched on top of a dandelion without deforming the soft dandelion seeds, indicating the ultra-lightweight and low-density characteristics of the FCCF sample. Figure S6b shows that the FCCF-2 sample exhibits obvious magnetic properties, which is beneficial for improving the magnetic loss. Furthermore, the FCCF-2 sample could maintain its structure without mechanical damage even under a load of 3000 times its own weight (Fig. S6c), owing to its excellent structural robustness. As shown in Fig. 4k, when the 5-mm-thick FCCF-2 sample is placed on the heating platform with the temperature set at 100, 120, and 150 °C, respectively, the surface temperatures of the FCCF-2 sample are only 65, 71, and 86 °C, respectively. Moreover, the surface temperature of FCCF-2 remains stable at around 60 °C as the heating time is increased (Fig. S7). Therefore, it is considered that the FCCF-2 sample possesses excellent thermal insulating performance. Furthermore, the Joule heating performance of the FCCF-2 sample is investigated by applying a voltage to both ends of the foam. The infrared thermal images shown in Fig. 4l intuitively indicate the surface temperature of the FCCF-2 sample under different voltages. At voltages of 5–25 V, the FCCF-2 sample exhibits excellent Joule heating performance with surface temperatures ranging from 28.5 to 98.4 °C. It is believed that the 3D interconnected CNC-CF network provides efficient paths for electron transfer, which endows the FCCF-2 sample with excellent electrothermal conversion capability. In a word, the excellent thermal insulation is essential to protect sensitive electronic equipment from high temperatures. Joule heating performance also protects equipment from freezing environments. Therefore, we believe that the composite carbon foam absorbers have the potential to cope with the harsh practical application environment [66–68].

### Microwave Absorption Mechanisms of the Samples

In Fig. 5, the electromagnetic parameters of CF, CCF, FCF, and FCCF-2 samples are further analyzed to investigate the dielectric microwave dissipation mechanisms of the sample. It is shown in Fig. 5a, b that the CF sample exhibits the lowest $\varepsilon^{\prime }$ and $\varepsilon^{\prime \prime }$ values, indicating the weak dielectric loss of the CF sample. For the CCF sample, the introduction of the CNCs greatly improves the $\varepsilon^{\prime }$ and $\varepsilon^{\prime \prime }$ values, which endows the CCF sample with strong dielectric loss. However, the excessive $\varepsilon^{\prime }$ and $\varepsilon^{\prime \prime }$ values result in poor impedance matching (Fig. 3h). Thus, the moderate $\varepsilon^{\prime }$ and $\varepsilon^{\prime \prime }$ values endow the FCF and FCCF-2 samples with considerable dielectric loss and good impedance matching. As shown in Fig. S8a, the dielectric loss tangent curves of the samples are calculated. It is observed that the CCF sample exhibits the strongest dielectric loss capability. However, the poor impedance matching of the CCF sample limits the microwave absorption performance. The FCF and FCCF-2 samples both possess strong dielectric loss capability and good impedance matching, resulting in the excellent microwave absorption performance. Therefore, the synergistic effect between the dielectric loss and impedance matching is beneficial for achieving excellent microwave absorption performance. Moreover, the Raman spectra (Figs. 5c and S2c) of the samples all exhibit two characteristic peaks, D-band and G-band peaks, at 1340.1 and 1580.8 cm^−1^, respectively, indicating the graphitic structures of the samples. In general, the intensity ratio of D-band to G-band (*I*_D_/*I*_G_) stands for the graphitization degree of the graphitic structures [69–72]. It is observed in Figs. 5c and S2c that the CCF and FCCF samples show lower *I*_D_/*I*_G_ values compared to the CF and FCF samples, confirming that the introduction of the CNCs promotes the graphitization degree and enhances the electron transfer capability. For the CCF sample, the growth of the CNCs endows the CCF sample with a dense conductive network, resulting in an excessive electron transport capability. The introduction of magnetic nanoparticles further reduces the electron transport capability of the conductive network. However, in Fig. 5c, the high *I*_D_/*I*_G_ value of the CF sample indicates the poor graphitization degree. Thus, the electron transport capability of the CF sample is extremely weak. In this situation, the crystal structures of the magnetic particles improve the electron transport capability of the CF sample instead. Therefore, the introduction of the FeNi/NiFe_2_O_4_ particles contributes to the moderate $\varepsilon^{\prime }$ and $\varepsilon^{\prime \prime }$ values. In addition, the Cole–Cole curves of the samples are calculated to further investigate the polarization loss of the samples according to the Debye relaxation theory [73–76]:

4 $$ \left( {\varepsilon^{\prime} - \frac{{\varepsilon_{s} + \varepsilon_{\infty } }}{2}} \right)^{2} + \left( {\varepsilon^{\prime\prime}} \right)^{2} = \left( {\frac{{\varepsilon_{s} + \varepsilon_{\infty } }}{2}} \right)^{2} $$

where $\varepsilon_{s}$ and $\varepsilon_{\infty }$ represent the static permittivity and the relative permittivity at the high-frequency limit, respectively. As presented in Fig. 5d, the CF sample exhibits several semicircles, indicating the existence of multiple polarization processes. Due to the low graphitization degree of CF samples, these polarization processes are attributed to the dipole polarization induced by the defects and functional groups. For the CCF sample, the promoted graphitization degree and the CNC-CF conductive network induce strong conduction loss. Thus, the Cole–Cole curve of the CCF sample shows a linear shape. For FCF and FCCF-2 samples, the electron transfer capability is further adjusted by the FeNi-based nanoparticles. Moreover, the formation of the metal–carbon heterointerfaces induces interfacial polarization. The Raman spectra results show that the FCF and FCCF-2 samples both contain numerous defects, which induce the dipole polarization. Therefore, the Cole–Cole curves of the FCF and FCCF-2 samples show the conduction loss and polarization loss simultaneously. According to the Debye relaxation theory, the transfer of the electrons results in the distortion of the Cole–Cole semicircle. In Fig. 5f, g, the approximately linear curve means that the distortion of the semicircle was at a high level. Thus, conduction loss is the main loss mechanism in dielectric loss. As illustrated in Fig. 5h–k, the dielectric loss mechanisms are presented in accordance with the aforementioned results. Firstly, the main dielectric loss mechanism of the CF sample is dipole polarization due to the poor graphitization degree (Fig. 5h). Secondly, the conduction loss [26] is considered as the main dielectric loss mechanism of the CCF sample due to the formation of the CNC-CF conductive network (Fig. 5i, j). Finally, the introduction of FeNi-based nanoparticles induces interfacial polarization, which is further confirmed by the density functional theory (DFT) calculations (Figs. 5k and S9). Figure 5k provides the calculated electron density difference and corresponding isosurface plot, in which the yellow and cyan regions denote electron density accumulation and depletion, respectively. It is observed that the charges accumulate at the heterointerface between carbon and FeNi, which induce the interfacial polarization. Moreover, the number of transferred electrons at the carbon-FeNi interface is calculated (Fig. S10), further confirming the existence of the interfacial polarization at the heterointerface between carbon and FeNi. It is reasonable to conclude that the dielectric loss mechanism of FCCF-2 composites could be attributed to the synergistic effect of the conduction loss and polarization loss.

**Fig. 6:**
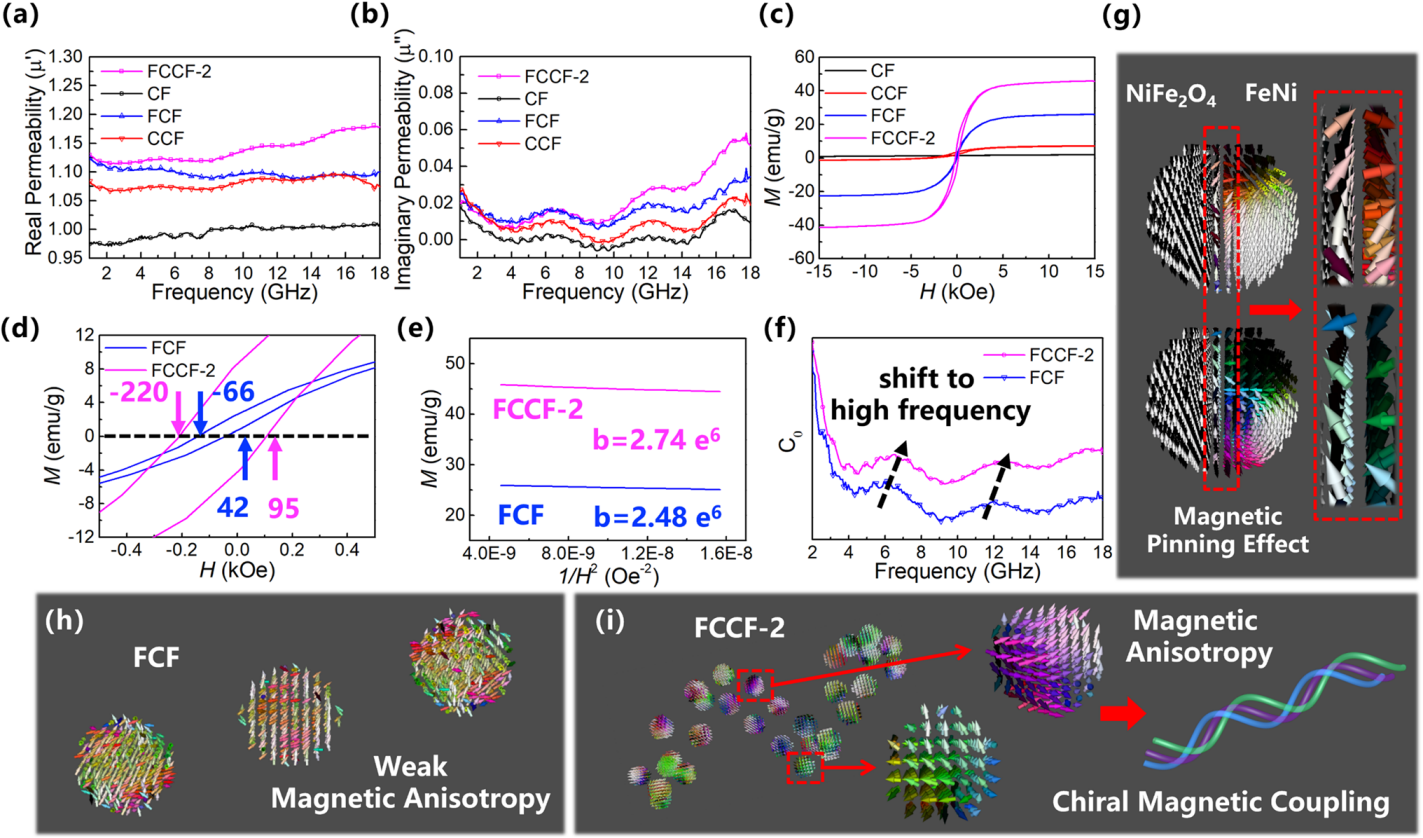
**a** Real permeability, **b** imaginary permeability, and **c** M-H curves of CF, CCF, FCF, and FCCF-2 samples; **d** Enlarged M-H curves, **e** M versus 1/H^2^ plots, and **f** eddy current induction coefficient of FCF and FCCF-2 samples; micromagnetic simulation of **g** NiFe_2_O_4_-FeNi magnetic heterostructures, **h** linear FCF sample, and **i** chiral FCCF-2 sample

The permeability of the samples is compared in Fig. 6a, b to further investigate their magnetic loss mechanisms. It is observed that the $\mu^{\prime }$ and $\mu^{\prime \prime }$ values of the CF and CCF samples are close to 1 and 0, respectively, due to their weak magnetic property. Moreover, the FCF samples exhibit higher $\mu^{\prime }$ and $\mu^{\prime \prime }$ values due to the formation of the magnetic FeNi-NiFe_2_O_4_ particles. More importantly, the FCCF-2 sample shows the highest $\mu^{\prime }$ and $\mu^{\prime \prime }$ values, indicating that the chiral magnetic structure and the nanoscale magnetic heterostructure are beneficial to improve the magnetic loss. In addition, the room-temperature magnetic hysteresis loops (Fig. 6c) are tested to confirm the excellent intrinsic magnetic properties of the FCCF-2 sample. It is observed that the FCCF-2 samples exhibit the highest saturation magnetization (*M*_*s*_) values, which is consistent with the variation tendency of the $\mu^{\prime }$ and $\mu^{\prime \prime }$ curves. Figure S2d also shows that the *M*_*s*_ values of the FCCF samples could be well adjusted by modulating the growth density of the magnetic nanoparticles. In the CNC-carbon foam network, the CNCs act as the branches of the carbon foam trunk, increasing the surface area of the sample and providing more growth sites for the magnetic nanoparticles. Therefore, the FCCF-2 sample has a higher saturation magnetization value than the FCF sample. Besides, Figs. 6d and S11 show that the FCF and FCCF samples both show the asymmetry magnetic hysteresis loops, indicating the existence of the exchange bias induced by the FeNi-NiFe_2_O_4_ magnetic heterostructures. According to the research of Wang et al. [12], the exchange bias originates from the magnetic pinning effect at the interface of the magnetic heterostructures, which is conducive to increasing the magnetocrystalline anisotropy field (*H*_*k*_). Generally, the *H*_*k*_ could be estimated by comparing the hysteresis loops of the sample using the simplified formulas of the S-W approximation [77, 78]:

5 $$ M = M_{s} \left( {1 - \frac{b}{{H^{2} }}} \right),H_{k} {\text{ = c}} \cdot b^{\frac{1}{2}} $$

In the above formulae, c is a constant and *b* is the slope of the M-1/H^2^ plots. As shown in Fig. 6e, the slope *b* of the FCCF-2 sample is greater than that of the FCF sample, which indicates that the FCCF-2 sample possesses the larger *H*_*k*_. According to the Snoke’s limit, the larger *H*_*k*_ gives rise to the larger natural resonance frequency, which is beneficial for improving the magnetic loss. Thus, the eddy current induction coefficient *C*_0_ of the FCF and FCCF-2 samples is calculated ulteriorly to investigate the magnetic resonance [79–82]:

6 $$ C_{0} { = }\mu^{\prime\prime}\left( {\mu^{\prime}} \right)^{ - 2} \left( f \right)^{ - 1} $$

In general, the fluctuations in the *C*_0_ curves indicate the existence of magnetic resonance. According to the ferromagnetic resonance theory, the resonance peaks appearing at 2–10 GHz represent the natural resonance, while the resonance peaks at 10–18 GHz stand for the exchange resonance. As shown in Fig. S8b, the magnetic loss tangent curves of the FCF and FCCF-2 samples are calculated. It is observed that the magnetic loss tangent curves of the FCF and FCCF-2 samples also show several resonance peaks, which is consistent with the *C*_0_ curves. In Figs. 6f and S8b, the FCF and FCCF-2 samples both exhibit natural and exchange resonances. However, the FCCF-2 sample exhibits a higher natural resonance frequency, further confirming that the FCCF-2 sample possesses the larger *H*_*k*_ and promotes the Snoke’s limit efficiently.

Furthermore, the dynamic evolution processes of the magnetic domains are explored by the micromagnetic simulations to confirm the magnetic loss mechanisms of the FCF and FCCF-2 samples. Firstly, a magnetic heterostructure composed of the ferrimagnetic NiFe_2_O_4_ and ferromagnetic FeNi is constructed to confirm the magnetic pinning effect. In Fig. 6g, a magnetic heterostructure composed of the ferrimagnetic NiFe_2_O_4_ and ferromagnetic FeNi is constructed to confirm the magnetic pinning effect. For the ferromagnetic FeNi, the external alternating magnetic field results in the rotation of magnetic moments. Thus, at the heterointerface between the NiFe_2_O_4_ and FeNi, the magnetic moment of the ferromagnetic FeNi generates an exchange magnetic field, acting on the uncompensated magnetic moment of the ferrimagnetic NiFe_2_O_4_. In contrast, the uncompensated magnetic moments around the interface in the ferrimagnetic NiFe_2_O_4_ also hinder the rotation of magnetic moments in the ferromagnetic FeNi. Therefore, the magnetic loss capability is enhanced by the magnetic pinning effect. Moreover, the microscale linear structure of the FCF sample is constructed by linearly arranging the microscale magnetic particles (Fig. 6h), where the color of the magnetic moments represents the orientations. In Fig. 6h, the multicolor distribution of the FCF sample stands for the chaotic magnetic moment orientations, indicating the weak magnetic anisotropy and weak magnetic coupling. Therefore, the magnetic loss mechanism of the FCF sample is attributed to the rotation of magnetic moments. Moreover, the nanoscale chiral structure of the FCCF-2 sample is also constructed (Fig. 6i). As the magnetic particle size decreases, the magnetic domain orientations tend to be oriented in the same direction, indicating the enhanced magnetic anisotropy. In addition, the magnetic domains in different helical directions show an obvious magnetic coupling effect. In our previous research [29], the enhanced magnetic coupling effect was studied in detail. For the chiral helical structure, the magnetic coupling effects occur not only along the axis of the helical fiber, but also between the helical rings. Thus, it is concluded that the chiral structures enhance the magnetic loss capability of the FCCF-2 sample. In a word, the nanoscale chiral magnetic heterostructures in the FCCF-2 sample achieve strong magnetic anisotropy and high magnetic loss, which contribute to the excellent microwave absorption performance.

In addition, the quarter-wavelength matching model [30] is introduced to better analyze the microwave absorption mechanisms of the samples:

7 $$ t_{m} = \frac{n\lambda }{4} = \frac{nc}{{4f_{m} }}\frac{1}{{\sqrt {\left| {\varepsilon_{{\text{r}}} } \right|\left| {\mu_{{\text{r}}} } \right|} }};\,\,n = 1,3,5, \cdots $$

In the above formulae, the $t_{m}$ represents the thickness of the absorber, and the $f_{m}$ stands for the peak frequency of the RL value. Generally, if the $t_{m}$ and the $f_{m}$ accord well with the model, the phase cancelation effect would occur to largely reduce the reflection of the microwave. In Fig. S12, the quarter-wavelength matching model curves of the samples are calculated and depicted as the blue dots. It is observed that all the samples accord well with the quarter-wavelength matching model, indicating that the phase cancelation effect contributes to their microwave absorption performance. In addition, Fig. S13 shows that the regions where the impedance matching values are close to 1 also accord well with the quarter-wavelength matching model. Therefore, it is concluded that the coexistence of the quarter-wavelength matching models and the impedance matching is necessary to obtain the strong microwave absorption performances.

## Conclusions

In summary, the chiral CNCs are first synthesized on a 3D carbon foam and then combined with the FeNi/NiFe_2_O_4_ nanoparticles to form a novel chiral-dielectric-magnetic trinity foam via chemical vapor deposition (CVD) and solvothermal reactions. The efficient 3D CNC-CF conductive network provided strong conduction loss, and the formation of metal–carbon interface induced interfacial polarization loss. The nanoscale chiral magnetic heterostructures exhibited magnetic pinning and magnetic coupling effects, further enhancing the magnetic anisotropy and magnetic loss capability. Owing to the synergistic effect between dielectricity, chirality, and magnetism, the trinity composite foam exhibits excellent microwave absorption performance with an ultrabroad EAB of 14 GHz and a minimum reflection of loss less than − 50 dB. More importantly, the C-band EAB of the foam is extended to 4 GHz, achieving the full C-band coverage. These results can be used as the guidelines for the design of efficient chiral microwave absorbers.

## Supplementary Information

Below is the link to the electronic supplementary material.

Supplementary file1 (DOCX 7444 KB)
