# Supplementary material for: Multifunctional Carbon Foam with Nanoscale Chiral Magnetic Heterostructures for Broadband Microwave Absorption in Low Frequency
Source: Nanomicro Lett. 2025 Feb 6;17:133. doi: 10.1007/s40820-025-01658-8 (PMC11799491; doi:10.1007/s40820-025-01658-8)
Supplement: Supplementary file 1 — Supplementary file1 (DOCX 7444 KB) [file 40820_2025_1658_MOESM1_ESM.docx]

Supporting Information for

**Multifunctional Carbon Foam with Nanoscale Chiral Magnetic Heterostructures for Broadband Microwave Absorption in Low Frequency**

Hao Zhang^1^, Kaili Kuang^1^, Yifeng Zhang^1^, Chen Sun^1^, Tingkang Yuan^1^, Ruilin Yin^1^, Zeng Fan^1^, Renchao Che^2,^ * and Lujun Pan^1,^ *

^1^ School of Physics, Dalian University of Technology, Dalian, Liaoning 116024, P. R. China

^2^ Laboratory of Advanced Materials, Shanghai Key Lab of Molecular Catalysis and Innovative Materials, Department of Materials Science, Fudan University, Shanghai 200438, P. R. China

*Corresponding authors. E-mail: [lpan@dlut.edu.cn](mailto:lpan@dlut.edu.cn) (Lujun Pan), [rcche@fudan.edu.cn](mailto:rcche@fudan.edu.cn) (Renchao Che)

**Supplementary Figures and Tables**


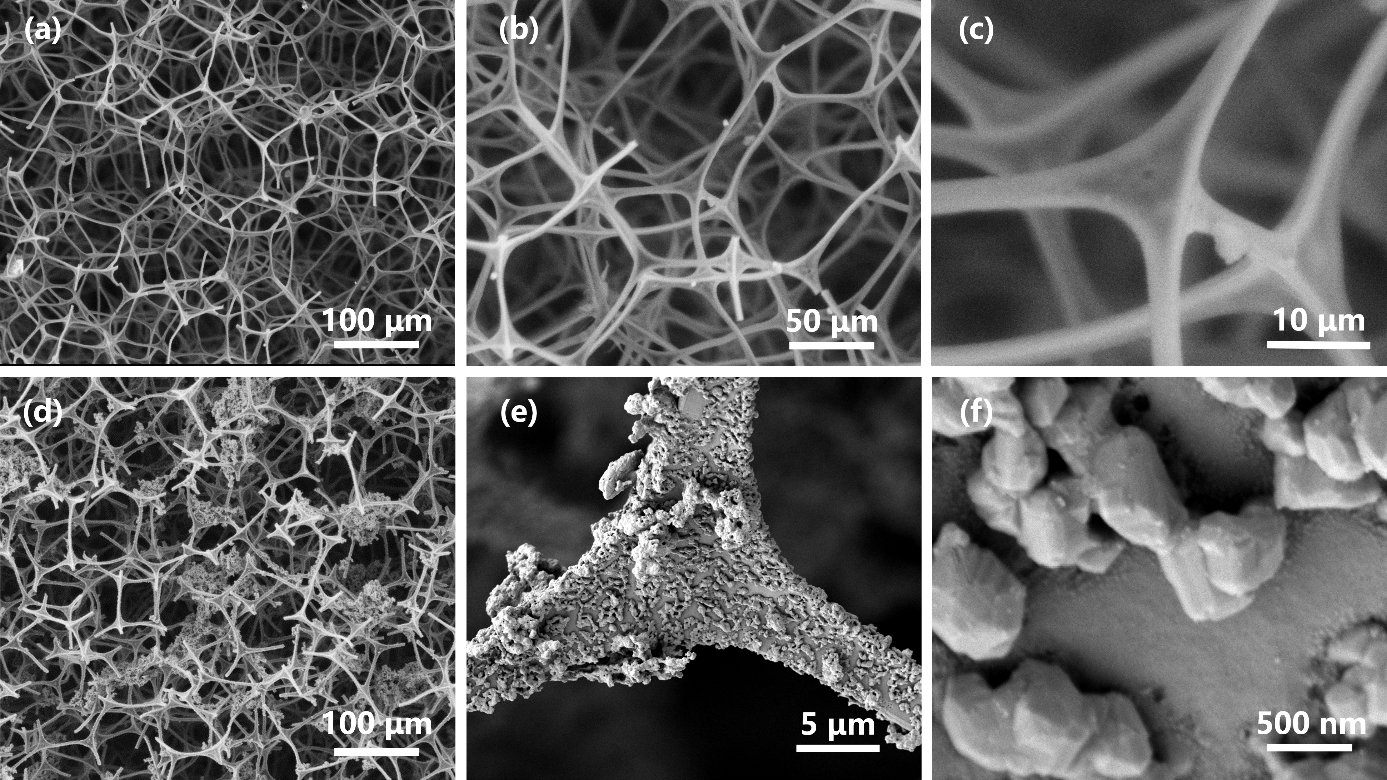


**Fig. S1** SEM images of (**a-c**) CF samples and (**d-f**) FCF samples


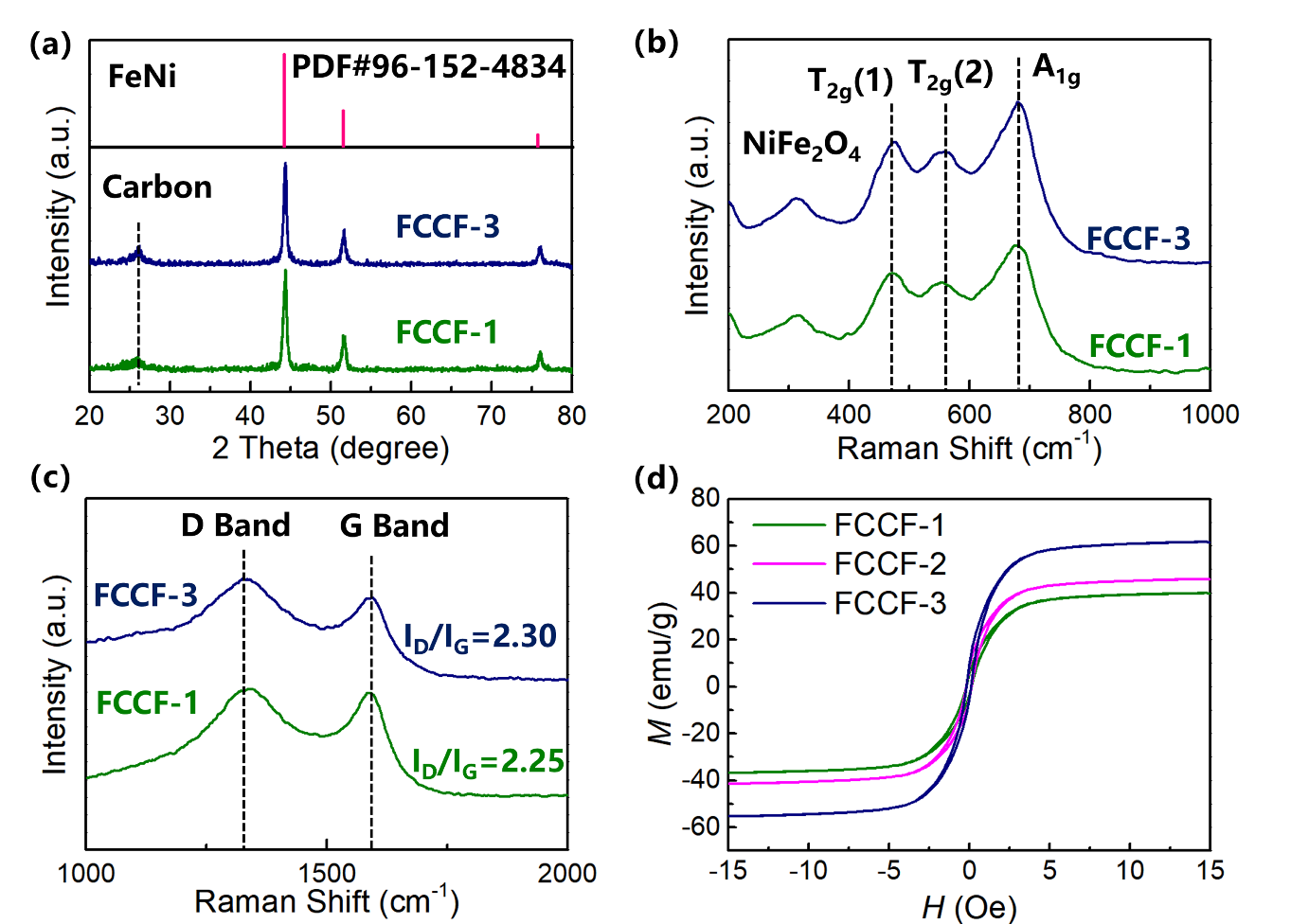


**Fig. S2** (**a**) XRD patterns, (**b, c**) Raman spectra, and (**d**) magnetic hysteresis loops of the FCCF-1 and FCCF-3 samples


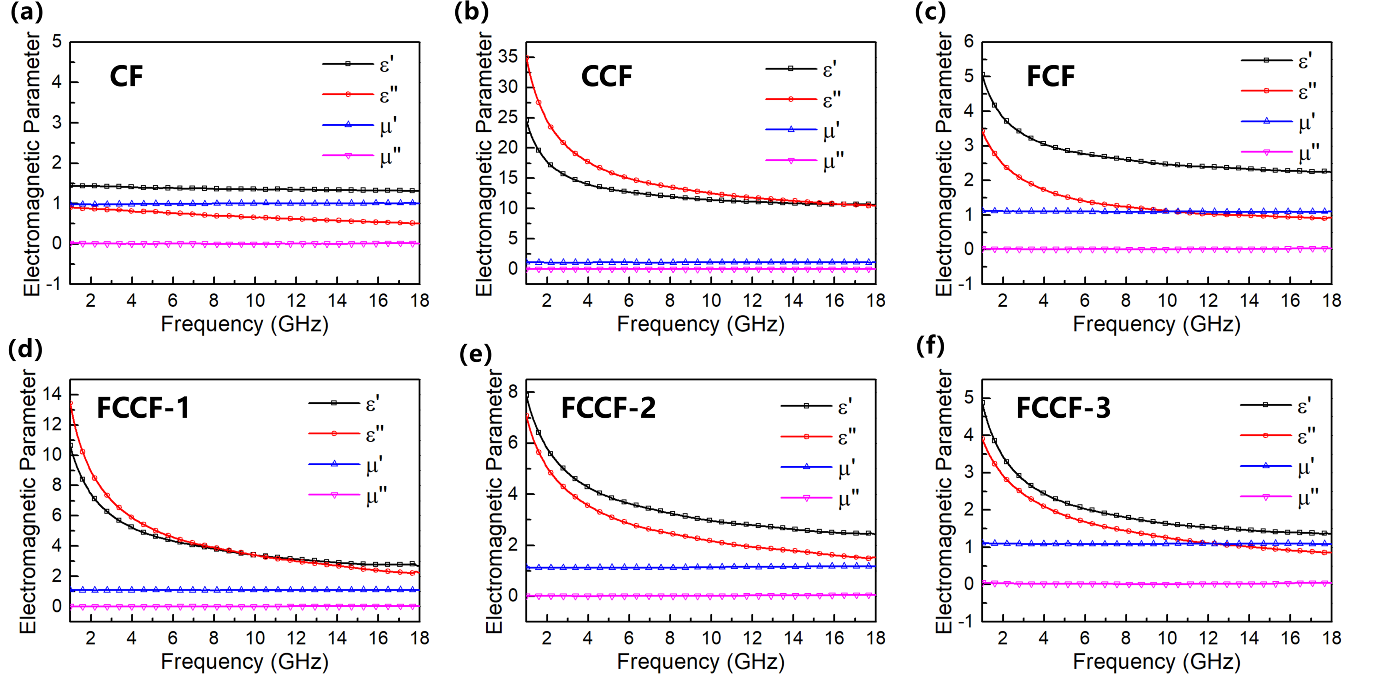


**Fig. S3** Electromagnetic parameters of (**a**) CF composites, (**b**) CCF composites, (**c**) FCF composites, (**d**) FCCF-1 composites, (**e**) FCCF-2 composites and (**f**) FCCF-2 composites


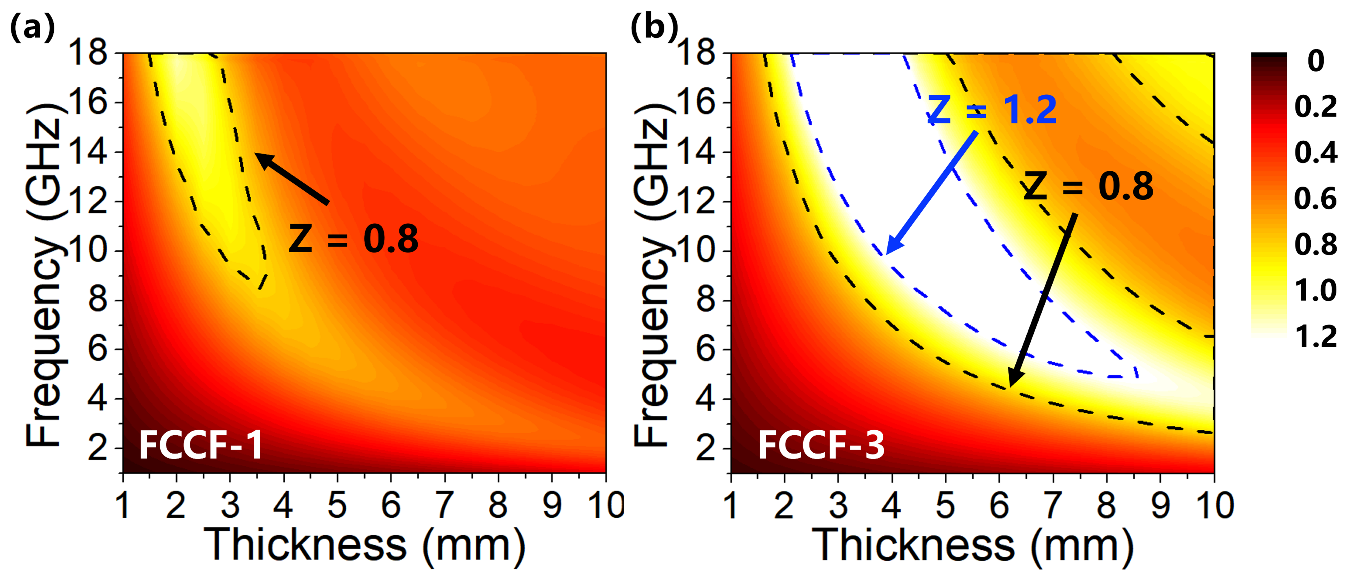


**Fig. S4** Impedance matching contour maps of (**a**) FCCF-1 and (**b**) FCCF-3 samples


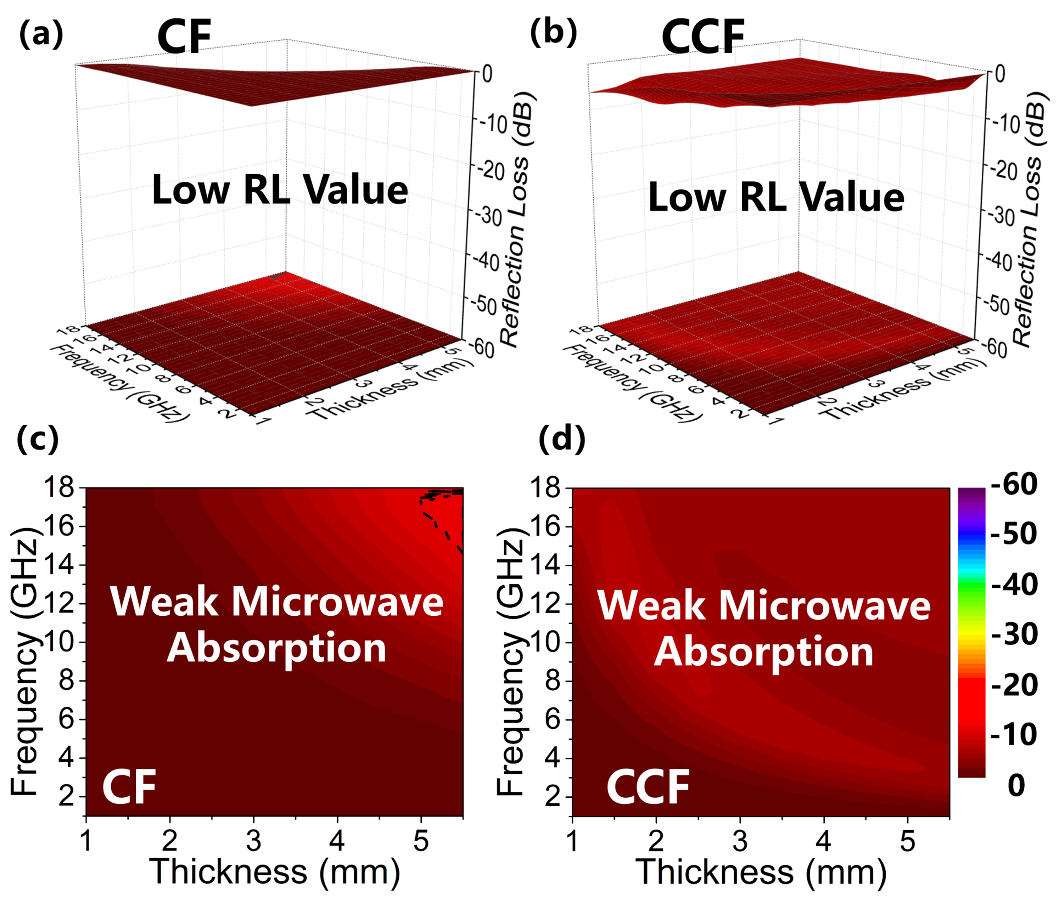


**Fig. S5** 3D RL and 3D projection plots of (**a, c**) CF and (**b, d**) CCF samples


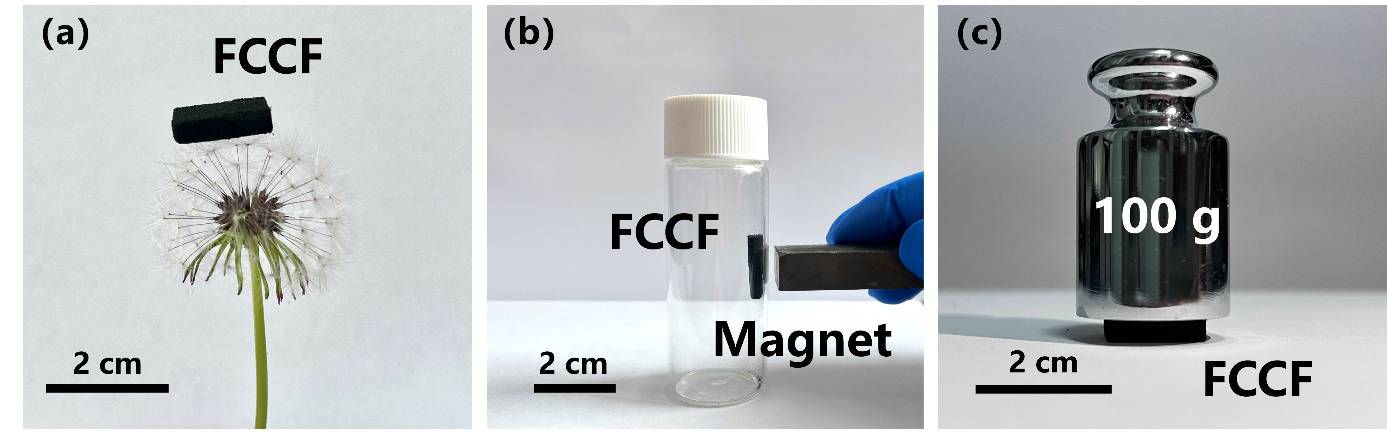


**Fig. S6** (**a**) Optical image, (**b**) Magnetic properties, and (**c**) Structural robustness of the FCCF samples


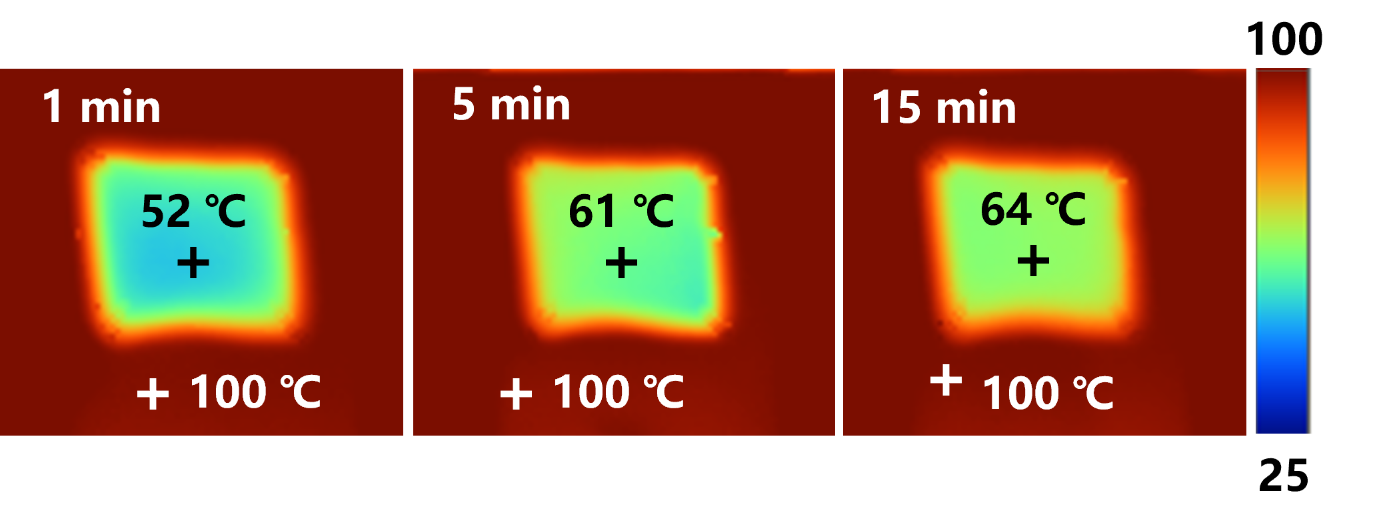


**Fig. S7** Infrared radiation images of surface temperature of FCCF-2 sample at different times on a platform with the temperature of 150 °C


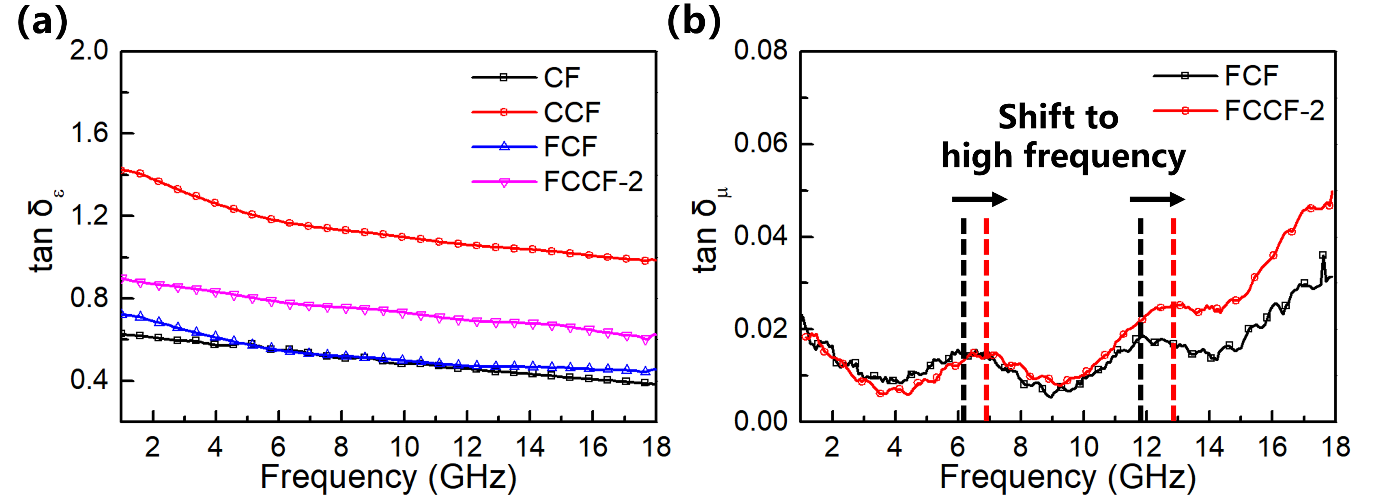


**Fig. S8** (**a**) Dielectric loss tangent curves and (**b**) Magnetic loss tangent curves of the samples


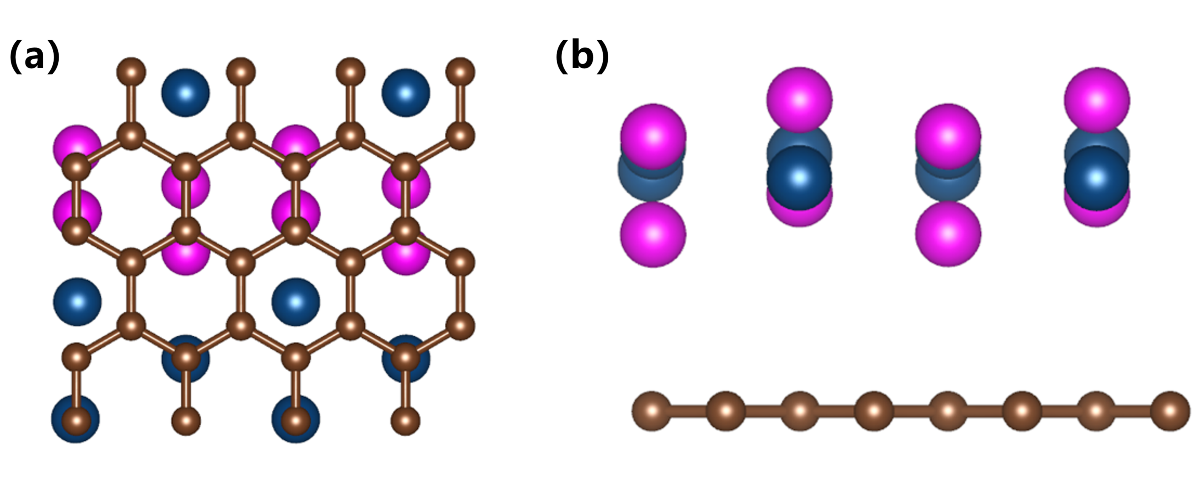


**Fig. S9** (**a**) Top and (**b**) side views of C/FeNi DFT models


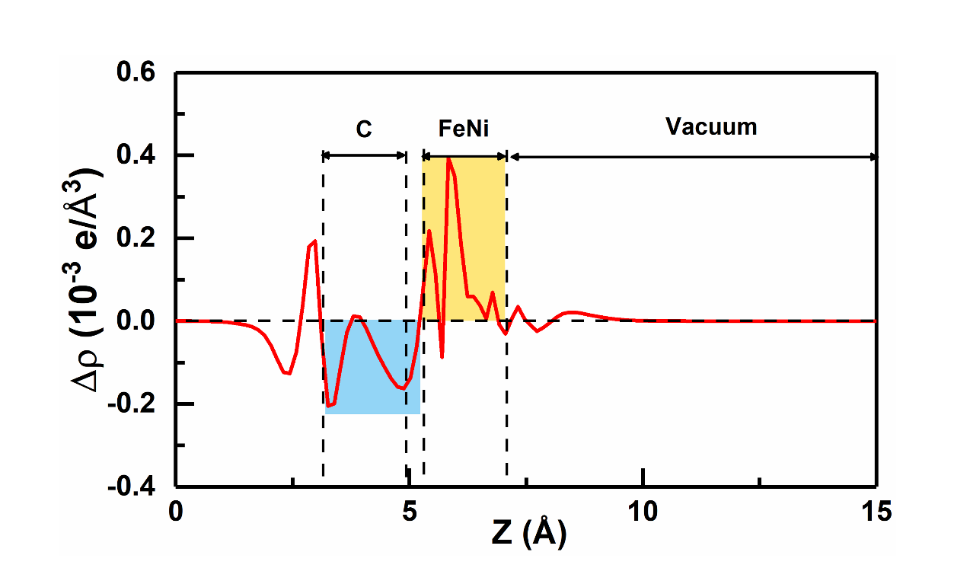


**Fig. S10** Integration of ρ in planes parallel to the surface and plotted as a function of the z coordination


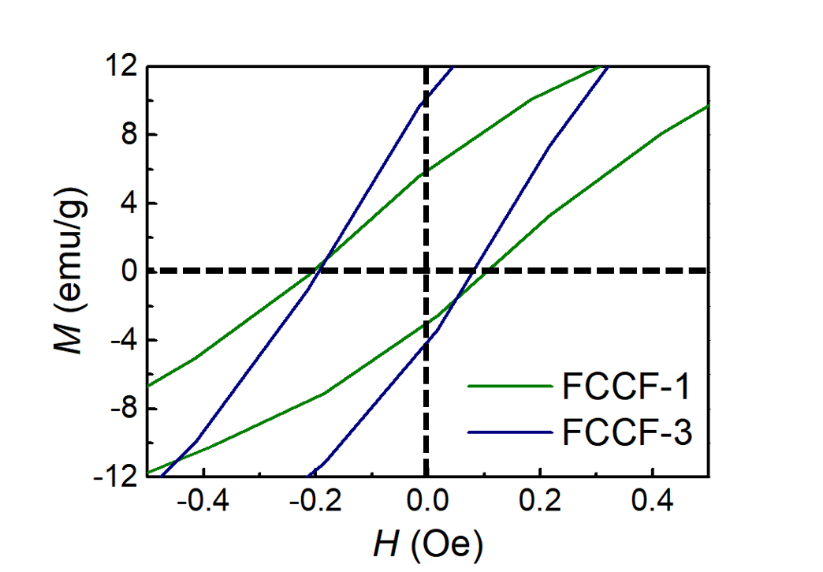


**Fig. S11** Enlarged magnetic hysteresis loops of the FCCF-1 and FCCF-3 samples


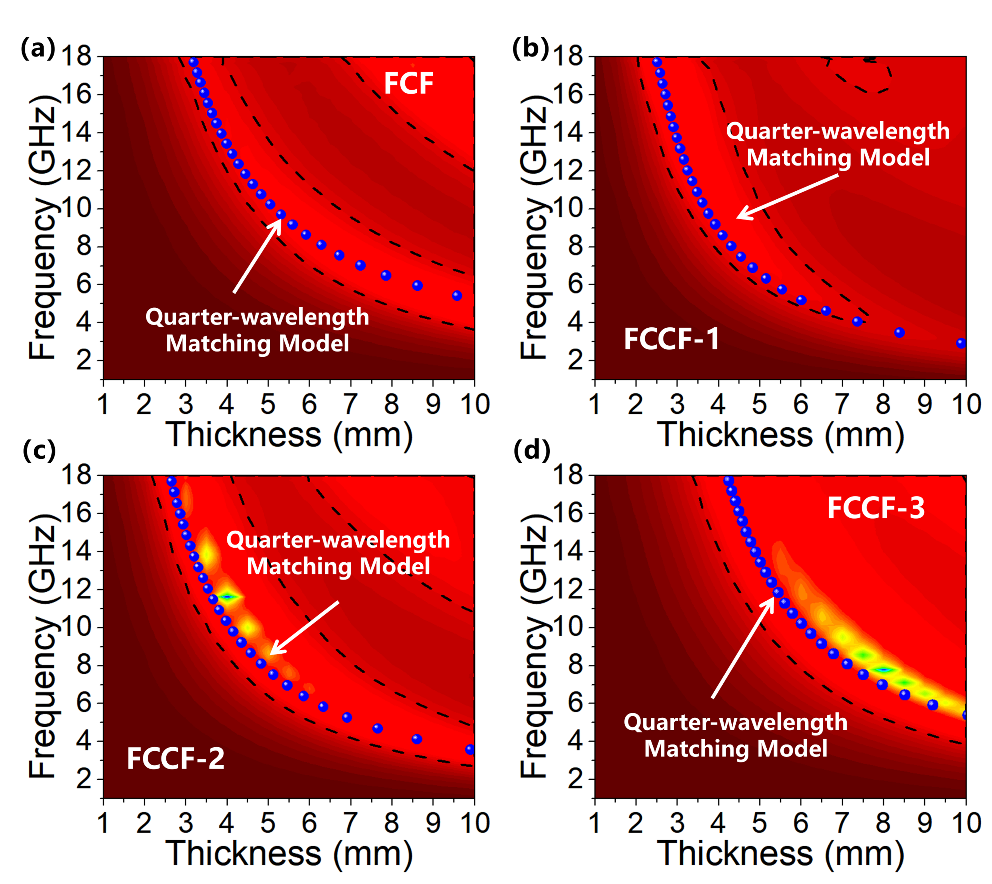


**Fig. S12** Reflection loss contour maps with quarter-wavelength matching curves of the samples


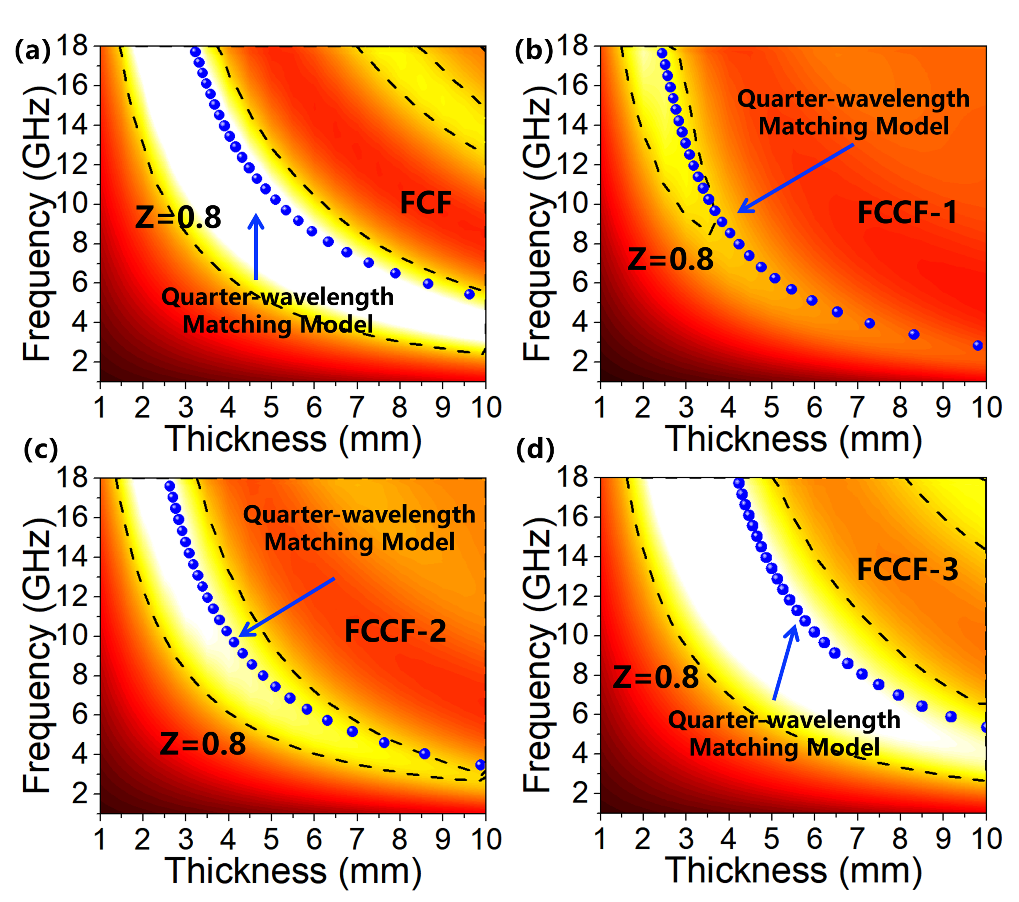


**Fig. S13** Impedance matching contour maps with quarter-wavelength matching curves of the samples


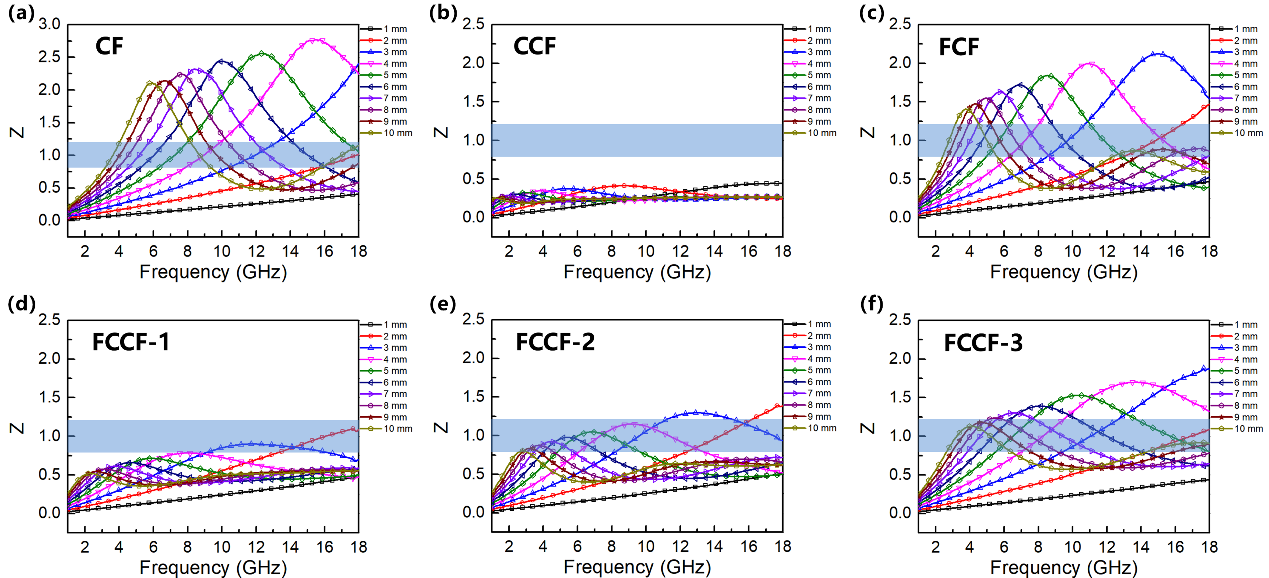


**Fig. S14** 2D impedance matching images of the samples


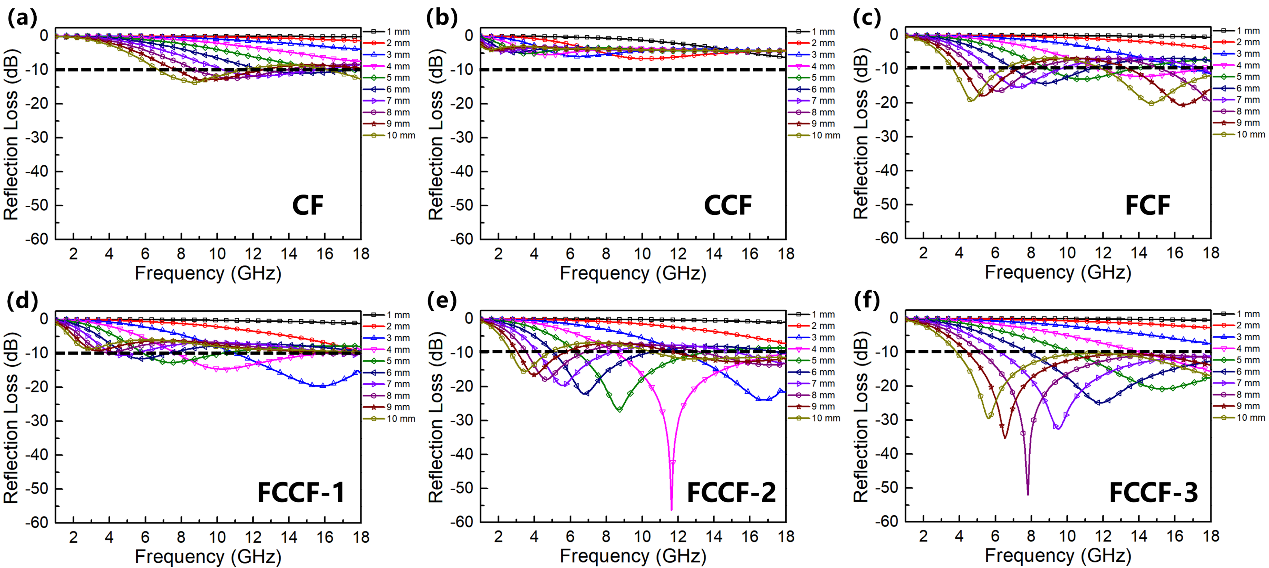


**Fig. S15** 2D reflection loss images of the samples

**Table S1** Detailed information on the concentration of the composites for electromagnetic measurement

| Sample | Fe^2+^  (mmol) | Ni^2+^  (mmol) | HMT  (mmol) | Solvent  (H_2_O/C_2_H_5_OH) | Annealing  Temperature (℃) |
| --- | --- | --- | --- | --- | --- |
| FCF | 1.5 | 3 | 6 | 20 mL/20 mL | 700 |
| FCCF-1 | 1 | 2 | 4 | 20 mL/20 mL | 700 |
| FCCF-2 | 1.5 | 3 | 6 | 20 mL/20 mL | 700 |
| FCCF-3 | 2 | 4 | 8 | 20 mL/20 mL | 700 |

**Synthesis mechanisms of CCF and FCCF samples**

The synthesis mechanisms of CCF and FCCF are concluded as follows:

a. The CCF samples are prepared by growing CNCs on carbon foam (CF) via a CVD process. First, a type of efficient bimetallic catalyst (α-Fe_2_O_3_/SnO_2_) in the form of nanoparticles is loaded onto carbon foam by a dip coating process. The α-Fe_2_O_3_/SnO_2_/CF samples are preheated to 710 °C in an Ar atmosphere for 30 min. The CNCs are then synthesized on the CF samples with the introduction of additional C_2_H_2_ gases at 710 °C. During the CNC growth process, Fe-containing particles are the main catalytic components in the catalyst system, decomposing C_2_H_2_ and depositing a carbon filament. The presence of Sn and SnO_2_ reduces the catalytic activity of Fe-containing particles. The mechanism of CNC growth is attributed to the multiple particles at the tip of a CNC with different catalytic activities corresponding to different growth rates of the carbon filaments. In our previous work (*Nano-Micro Lett. (2020) 12:23; Carbon 166 (2020) 101-112*), the mechanism of CNC growth was investigated in detail.

b. The FCCF samples are prepared by growing FeNi-based nanoparticles on the CCF sample via a solvothermal reaction and a subsequent annealing treatment. In the solvothermal reaction, the hydrolysis of the hexamethylenetetramine (HMT) provides a mild basic environment (pH∼9). Hence, Ni^2+^ and Fe^2+^ couple with OH^-^, resulting in the formation of Ni(OH)_2_ and Fe(OH)_2_ structures on CCF sample. During the annealing treatment, the Ni(OH)_2_ and Fe(OH)_2_ structures are first transformed into NiFe_2_O_4_ structures, then the NiFe_2_O_4_ structures are partially reduced by carbon, resulting in the formation of the FeNi/NiFe_2_O_4_ structures. Following chemical equations explicitly interpreted the aforementioned reaction mechanism:

$C_{6}H_{12}N_{4}+H_{2}O\to CO_{2}+NH_{4}^{+}+OH^{-}$;

$Fe^{2+}+Ni^{2+}+OH^{-}\to Fe(OH)_{2}+Ni(OH)_{2}$;

$Fe(OH)_{2}+O_{2}+H_{2}O\to Fe(OH)_{3}$;

;

.

**Density Functional Theory (DFT) calculations**

The FeNi nanoparticles doped carbon model was constructed using first-principle calculation DFT via CP2K [S1-S6]. Diag procedure was used for the wave function optimization and the SCF convergence. The PBE functional [S7] was used with the D3 dispersion correction scheme [S8]. DZVP-MOLOPT-SR-GTH basis sets were used alongside planewaves expanded to a 600 Ry energy cutoff. Electronic cores were represented by Geodecker-Teter-Hutter pseudopotentials [S9, S10]. The 3s, 3p and 3d electrons of Fe/ Ni and 2s, 2p electrons of C are treated as valence. The k-point was set as 3×3×1. The periodic images were separated by vacuum layers 15 Å to eliminate image interactions.

**Micromagnetic Simulation Methods**

The micromagnetic simulation is performed by Mumax3 software, an open-source GPU-accelerated program. The space and time dependent magnetization dynamics in nano- to micro-sized ferromagnets can be calculated by the Mumax3 through the finite difference discretization. Based on the Landau-Lifshitz-Gilbert equation and the minimum energy principle, the finite difference algorithm simulates the dynamic physical process and solves the 3D model.

In this work, the dynamic spin structures of the FCCF-2 sample, FCF sample, and FeNi/NiFe_2_O_4_ heterostructures are simulated by the Mumax3. According to the VSM results, the saturation magnetizations of the magnetic particles in FCF and FCCF-2 samples are 2×10^5^ and 4×10^5^ A/m, respectively. The saturation magnetizations of the FeNi and NiFe_2_O_4_ in magnetic heterostructures are 4×10^5^ and 2×10^5^ A/m, respectively. The micromagnetic exchange constants of the magnetic particles in FCF and FCCF-2 samples are both 1.5×10^-11^ J/m. The micromagnetic exchange constants of the FeNi and NiFe_2_O_4_ in magnetic heterostructures are 1.5×10^-11^ and -1.5×10^-11^ J/m. The spin precession damping factor is 0.01. The frequency of the external magnetic field is 6 GHz. The diameters of the nanoparticles in FCCF-2 sample and FeNi/NiFe_2_O_4_ heterostructures are both 50 nm. The diameter of the nanoparticles in FCF sample is 500 nm.

**Supplementary References**

1. CP2K version 7.0 (Development Version), The CP2K developers group (2019). <https://www.cp2k.org/>
2. T.D. Kühne, M. Iannuzzi, M. Del Ben, V.V. Rybkin, P. Seewald et al., CP2K: an electronic structure and molecular dynamics software package - quickstep: efficient and accurate electronic structure calculations. J. Chem. Phys. **152**, 194103 (2020). <https://doi.org/10.1063/5.0007045>
3. J. VandeVondele, J. Hutter, An efficient orbital transformation method for electronic structure calculations. **118**, 4365–4369 (2003). <https://doi.org/10.1063/1.1543154>
4. J. VandeVondele, M. Krack, F. Mohamed, M. Parrinello, T. Chassaing et al., Quickstep: Fast and accurate density functional calculations using a mixed Gaussian and plane waves approach. Comput. Phys. Commun. **167**, 103–128 (2005). <https://doi.org/10.1016/j.cpc.2004.12.014>
5. J. Hutter, M. Iannuzzi, F. Schiffmann, J. VandeVondele, cp2k: atomistic simulations of condensed matter systems. Wiley Interdiscip. Rev. Comput. Mol. Sci. **4**, 15–25 (2014). <https://doi.org/10.1002/wcms.1159>
6. B.G. Lippert, J.H.A.M. Parrinello, A hybrid Gaussian and plane wave density functional scheme. mol Phys. **92**, 477–488 (1997). <https://doi.org/10.1080/002689797170220>
7. J.P. Perdew, K. Burke, M. Ernzerhof, Generalized gradient approximation made simple. Phys. Rev. Lett. **77**, 3865–3868 (1996). <https://doi.org/10.1103/PhysRevLett.77.3865>
8. S. Grimme, J. Antony, S. Ehrlich, H. Krieg, A consistent and accurate *ab initio* parametrization of density functional dispersion correction (DFT-D) for the 94 elements H-Pu. J. Chem. Phys. **132**, 154104 (2010). <https://doi.org/10.1063/1.3382344>
9. S. Goedecker, M. Teter, J. Hutter, Separable dual-space Gaussian pseudopotentials. Phys. Rev. B Condens. Matter **54**, 1703–1710 (1996). <https://doi.org/10.1103/physrevb.54.1703>
10. C. Hartwigsen, S. Goedecker, J. Hutter, Relativistic separable dual-space Gaussian pseudopotentials from H to Rn. Phys. Rev. B **58**, 3641–3662 (1998). <https://doi.org/10.1103/physrevb.58.3641>
